# The hard clam genome reveals massive expansion and diversification of inhibitors of apoptosis in Bivalvia

**DOI:** 10.1186/s12915-020-00943-9

**Published:** 2021-01-25

**Authors:** Hao Song, Ximing Guo, Lina Sun, Qianghui Wang, Fengming Han, Haiyan Wang, Gregory A. Wray, Phillip Davidson, Qing Wang, Zhi Hu, Cong Zhou, Zhenglin Yu, Meijie Yang, Jie Feng, Pu Shi, Yi Zhou, Libin Zhang, Tao Zhang

**Affiliations:** 1 CAS Key Laboratory of Marine Ecology and Environmental Sciences, Institute of Oceanology, Chinese Academy of Sciences, Qingdao, 266071 China; 2 Laboratory for Marine Ecology and Environmental Science, Qingdao National Laboratory for Marine Science and Technology, Qingdao, 266237 China; 3 Center for Ocean Mega-Science, Chinese Academy of Sciences, Qingdao, 266071 China; 4 CAS Engineering Laboratory for Marine Ranching, Institute of Oceanology, Chinese Academy of Sciences, Qingdao, 266071 China; 5 Haskin Shellfish Research Laboratory, Department of Marine and Coastal Sciences, Rutgers University, Port Norris, NJ USA; 6 University of the Chinese Academy of Sciences, Beijing, 100049 China; 7 Research and Development Center for Efficient Utilization of Coastal Bioresources, Yantai Institute of Coastal Zone Research, Chinese Academy of Sciences, Yantai, 264003 China; 8 Novogene Bioinformatics Institute, Beijing, 100029 China; 9 Duke University, 130 Science Dr, Durham, NC 27708 USA

**Keywords:** IAP gene family, Molecular evolution, Mollusca, Gene duplication, Divergence

## Abstract

**Background:**

Inhibitors of apoptosis (IAPs) are critical regulators of programmed cell death that are essential for development, oncogenesis, and immune and stress responses. However, available knowledge regarding IAP is largely biased toward humans and model species, while the distribution, function, and evolutionary novelties of this gene family remain poorly understood in many taxa, including Mollusca, the second most speciose phylum of Metazoa.

**Results:**

Here, we present a chromosome-level genome assembly of an economically significant bivalve, the hard clam *Mercenaria mercenaria*, which reveals an unexpected and dramatic expansion of the IAP gene family to 159 members, the largest IAP gene repertoire observed in any metazoan. Comparative genome analysis reveals that this massive expansion is characteristic of bivalves more generally. Reconstruction of the evolutionary history of molluscan IAP genes indicates that most originated in early metazoans and greatly expanded in Bivalvia through both lineage-specific tandem duplication and retroposition, with 37.1% of hard clam IAPs located on a single chromosome. The expanded IAPs have been subjected to frequent domain shuffling, which has in turn shaped their architectural diversity. Further, we observed that extant IAPs exhibit dynamic and orchestrated expression patterns among tissues and in response to different environmental stressors.

**Conclusions:**

Our results suggest that sophisticated regulation of apoptosis enabled by the massive expansion and diversification of IAPs has been crucial for the evolutionary success of hard clam and other molluscan lineages, allowing them to cope with local environmental stresses. This study broadens our understanding of IAP proteins and expression diversity and provides novel resources for studying molluscan biology and IAP function and evolution.

**Supplementary Information:**

The online version contains supplementary material available at 10.1186/s12915-020-00943-9.

## Background

Apoptosis or programmed cell death is essential to all multicellular animals in shaping organ formation during embryogenesis and later development [1–4], as well as regulating tissue homeostasis, elimination of damaged or abnormal cells, and mounting defense against infections [3, 5–9]. Reflecting these important functions, apoptosis is tightly regulated, and its dysregulation is associated with numerous pathologies. To counter various stimuli capable of triggering death, cells have devised sophisticated molecular machineries that guard against inappropriate or premature apoptosis [10]. Among these are inhibitor of apoptosis proteins (IAPs), which function primarily by suppressing caspases, the effector proteases in programmed cell death. Recent studies have illuminated some other vital roles played by IAPs, such as serving as transduction intermediates in signaling cascades associated with innate immune response, cell migration, and cell-cycle regulation [11–13].

The diverse functions performed by IAPs are driven, at least partially, by the diverse domain structure of the encoding gene family. The structure of IAPs has been extensively studied in mammals, where the IAP family consists of 10 members in seven types of diverse domain structures (Additional file 1: Fig. S1). IAPs are characterized by at least one baculoviral IAP repeat (BIR) domain, although the number of BIR domains and the presence of other domains are variable [14]. For example, both X-chromosome-linked IAP (XIAPs) and cellular IAPs (cIAPs) have 3 BIR domains in their N-terminal region, and a RING (really interesting new gene) finger domain at the C-terminal, while cIAPs have an extra caspase recruitment domain (CARD). X-linked IAPs can inhibit caspases by direct binding, while cIAPs bind to caspase-3 and caspase-7, without exerting direct inhibition, but marking them for proteasomal destruction [15, 16]. Also, cIAPs bind to tumor necrosis factor (TNF) receptor-associated factors (TRAFs), thereby blocking TNF receptor 1 (TNFR1)-induced cell death and promoting the activation of signaling pathways that induce the expression of pro-survival proteins [17]. Encoded by a single-copy gene in mammalian genomes, XIAP is the only IAP member that can directly inhibit caspases [18]. XIAP-null mice do not exhibit any pronounced phenotype [18], indicating the presence of a “back-up system” that can mitigate the loss of XIAP [19]. It is unclear how other IAPs function in complex biological pathways. The highly conserved BIR domain, its variation in number, and association with other domains highlight the structural and functional complexity of the IAP family.

Thus far, in-depth studies on IAPs and their functions are limited to a few model species from four metazoans groups: *Hydra vulgaris* of Cnidaria, *Caenorhabditis elegans* of Nematoda, *Drosophila melanogaster* of Insecta, and *Mus musculus* and *Homo sapiens* from Mammalia [8]. Mammalian IAPs have received maximum attention because of their importance to human health. Evasion of programmed cell death is a well-known hallmark of cancer, where IAPs play critical roles in attenuating apoptotic pathways by modulating the caspase cascade [20–22].

In contrast, IAPs have not been carefully examined in the vast majority of metazoan phyla including Mollusca, the second largest phylum of metazoans. Yet there are strong indications that IAPs may play critical roles in molluscs. The IAP gene family appears to have undergone expansion in the Pacific oyster *Crassostrea gigas*, and the expression of some IAPs is upregulated in response to biotic and abiotic stressors [23]. Large numbers of IAPs have also been reported in some other bivalves including *Bathymodiolus platifrons* [24], *Pinctada fucata* [25], and *Saccostrea glomerata* [26], although the expansion and evolution of IAPs in molluscs and many other protostomes are not well understood.

The hard clam *Mercenaria mercenaria* is a marine bivalve naturally distributed along the Atlantic coast of North America [27]. It lives in estuarine and nearshore sediments and can tolerate wide fluctuations in temperature and salinity [27–29]. Hard clam is a major aquaculture species and well-known for its “hardiness” and long shelf life out of seawater [27, 28]. To understand the diversity and evolution of molluscan IAPs, we produced a chromosome-level assembly of the hard clam genome and studied its IAP repertoire in comparison with other molluscs and non-molluscan metazoans. We also conducted transcriptomic studies to assess possible roles of hard clam IAPs in stress response. Our analyses reveal a massive expansion of IAPs in molluscs, particularly in bivalves and largest in hard clam, accompanied with remarkable structural and functional diversity that may be essential for stress response and adaptation in molluscs and other metazoans.

## Results

### Genome sequencing, assembly, and characterization

**Fig. 1:**
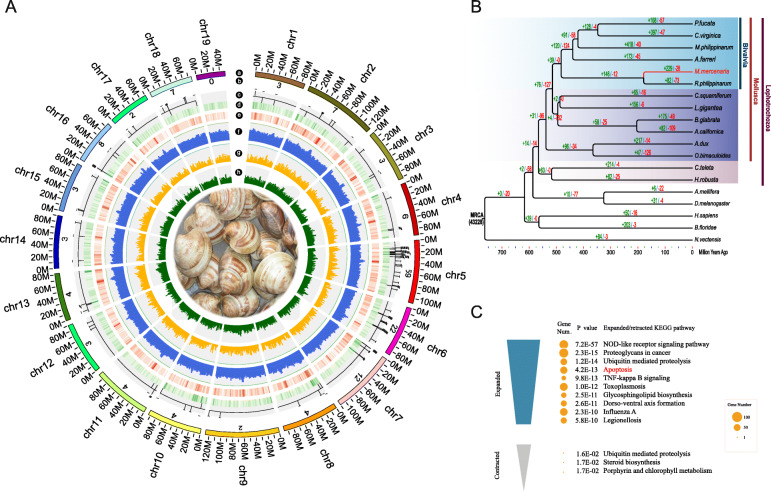
Characterization and phylogenetic analysis of the hard clam genome. **a** Genomic landscape of the hard clam. From outer to inner circles: a, the 19 chromosomes at the Mb scale; b, IAP gene number on each chromosome; c, chromosomal distribution of 159 IAPs, with outer and inner lines indicating sense and antisense strands, respectively. Each hyphen on circle margin represents an IAP gene copy; d~h, DNA transposons density, TE density, repeat density, gene density, and GC density across the genome, respectively, drawn in 1 Mb non-overlapping windows. **b** Time-calibrated phylogenetic tree of the hard clam within metazoans. Numbers on the branches indicate the number of genes gained (+, green) and lost (−, red). Pfu, *Pinctada fucata*; Cvi, *Crassostrea virginica*; Mph, *Modiolus philippinarum*; Afa, *Azumapecten farreri*; Mme, *Mercenaria mercenaria*; Rph, *Ruditapes philippinarum*; Csq, *Chrysomallon squamiferum*; Lgi, *Lottia gigantea*; Bgl, *Biomphalaria glabrata;* Aca, *Aplysia californica*; Adu, *Architeuthis dux*; Obi, *Octopus bimaculoides*; Cte, *Capitella teleta*; Hro, *Helobdella robusta*; Ame, *Apis mellifera*; Dme, *Drosophila melanogaster;* Hsa, *Homo sapiens*; Bfl, *Branchiostoma floridae*; Nve, *Nematostella vectensis*. **c** The top 10 expanded and top three contracted pathways that were enriched in the hard clam

The genome of the hard clam *M. mercenaria* was sequenced with Illumina reads, PacBio Single Molecule Real-Time (SMRT), 10X genomics, and Hi-C sequencing (Additional file 2: Table S1), resulting in a reference assembly of 1.79 GB with a contig N50 = 1.77 Mb (Additional file 2: Table S2). The size of the *M. mercenaria* genome was estimated as 1.78 GB (with 1.34% heterozygosity) based on *k*-mer analysis (Additional file 2: Table S3), which is close to the genome size estimated via flow cytometry [30]. Hi-C sequencing was used to position and orient 1541 scaffolds spanning 1.74 GB (scaffold N50 = 91.38 Mb) into 19 contiguous chromosomes, consistent with the haploid number (Fig. 1a, Additional file 4: Fig. S2 and Additional file 2: Table S4). The chromosome-level assembly was of high integrity and quality as over 95% of Illumina short-insert reads could be successfully mapped to the assembly (Additional file 2: Table S5). BUSCO assessment showed 90.5% completeness of the conserved core genes (Additional file 2: Table S6), which is comparable to or slightly lower (probably due to the hard clam’s large genome, high polymorphism, and the use of a non-inbred individual) than those of published bivalve genomes.

Annotation using EVidenceModeler combining ab initio prediction, homology to other species, and RNA-seq data identified 34,283 genes [31, 32] (Additional file 2: Table S7). The gene set of the hard clam is slightly larger than most bivalves sequenced to date, but similar to that of Eastern oyster *Crassostrea virginica* (34,596) and smaller than that of the mussel *Modiolus philippinarum* (36,549). In general, bivalve genomes encode more genes and show higher polymorphism than the human genome (Additional file 2: Table S8). Transposable elements (TEs) accounted for 49.11% of the genome with DNA transposon (34.24%), long terminal repeats (LTRs, 10.04%), and long interspersed elements (LINEs, 7.17%) comprising the 3 major transposon classes (Additional file 2: Table S9).

### Expansion of apoptosis-related domains and co-expression network

To determine the phylogenetic position of the hard clam, nineteen metazoan genomes (Fig. 1b) from Cnidaria, Annelida, Mollusca, Arthropoda, Chordata, and Vertebrata were selected for gene family clustering, which identified 43,245 gene families including 237 single-copy gene families. The single-copy genes from the 19 metazoan genomes were used for phylogenetic analysis with a maximum-likelihood method. We dated the divergence time of the hard clam from its nearest node (*Ruditapes philippinarum*) to approximately 178.9 Mya (Fig. 1b). From the common Heteroconchia ancestor to *M. mercenaria*, 229 gene families expanded with significant enrichment in genes related to immune response and apoptosis pathways such as NOD-like receptor signaling, ubiquitin-mediated proteolysis, and TNF-kappa B signaling (Fig. 1c, Additional file 3: Table S10). The expansion of immune and apoptosis-related gene families suggests that these gene families are important for the adaptation of the hard clam.

**Fig. 2:**
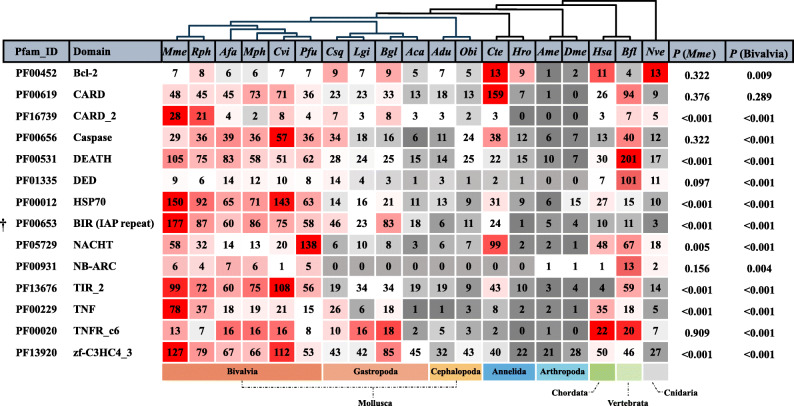
Distribution of protein Pfam domains associated with apoptosis in molluscs and other metazoans. Mme, *Mercenaria mercenaria*; Rph, *Ruditapes philippinarum*; Afa, *Azumapecten farreri*; Mph, *Modiolus philippinarum*; Cvi, *Crassostrea virginica*; Pfu, *Pinctada fucata*; Csq, *Chrysomallon squamiferum*; Lgi, *Lottia gigantea*; Bgl, *Biomphalaria glabrata;* Aca, *Aplysia californica*; Adu, *Architeuthis dux*; Obi, *Octopus bimaculoides*; Cte, *Capitella teleta*; Hro, *Helobdella robusta*; Ame, *Apis mellifera*; Dme, *Drosophila melanogaster;* Hsa, *Homo sapiens*; Bfl, *Branchiostoma floridae*; Nve, *Nematostella vectensis*. Domain abbreviations: Bcl-2, apoptosis regulator proteins, Bcl-2 family; CARD, caspase recruitment domain; DED, death effector domain; IAP, inhibitor of apoptosis domain; NACHT, a domain found in NAIP, CIITA, HET-E and TEP1 proteins; NB-ARC, a nucleotide-binding adaptor shared by APAF-1, certain R gene products, and CED-4; TIR, toll/interleukin-l receptor domain; TNF, tumor necrosis factor; TNFR, tumor necrosis factor receptor; zf-C3HC4_3, zinc finger, C3HC4 type (RING finger). Expansion in *M. mercenaria* or Bivalves is indicated by a significant, corrected *P* value (*P* < 0.001), from Chi-square tests for overrepresentation, using all annotated genes as background. Color, from gray to red, indicates ranking from bottom to top

Since apoptosis is one of the most significantly expanded pathway in *M. mercenaria*, we focused further analysis on the apoptosis pathway including regulatory signaling pathways such as NOD-like receptor signaling and TNF-kappa B signaling pathways. We searched apoptosis-related domains in the genome of *M. mercenaria* and compared our results with those obtained for other species (Fig. 2), to further characterize the expansion of genes related to apoptosis. Seven canonical domains related to apoptosis, BIR, TNF, DEATH, CARD2, TIR2 (toll/interleukin receptor 2), RING, and Hsp70 were greatly expanded in *M. mercenaria* and other bivalves (Fig. 2). The co-expansion of TNFs and BIRs in particular supports enhanced regulation of apoptosis, since in human, TNF and IAP (BIR containing protein) work together in regulating cell fate. For instance, TNF modulates diverse cellular responses, including apoptosis and necroptosis, via multiple signaling complexes originating from the TNFR superfamily, with cIAPs as an integral component, involving recruitment of downstream signal transducers [11–13]. The number of BIR domain-containing genes in hard clam, 177 copies, is the highest (*p* < 0.001) among all metazoans studied (Fig. 2). The significant expansion of domains related to apoptosis in hard clam and other bivalves suggests that bivalves may have a strong and complex gene set for regulating apoptosis and cellular stress.

**Fig. 3:**
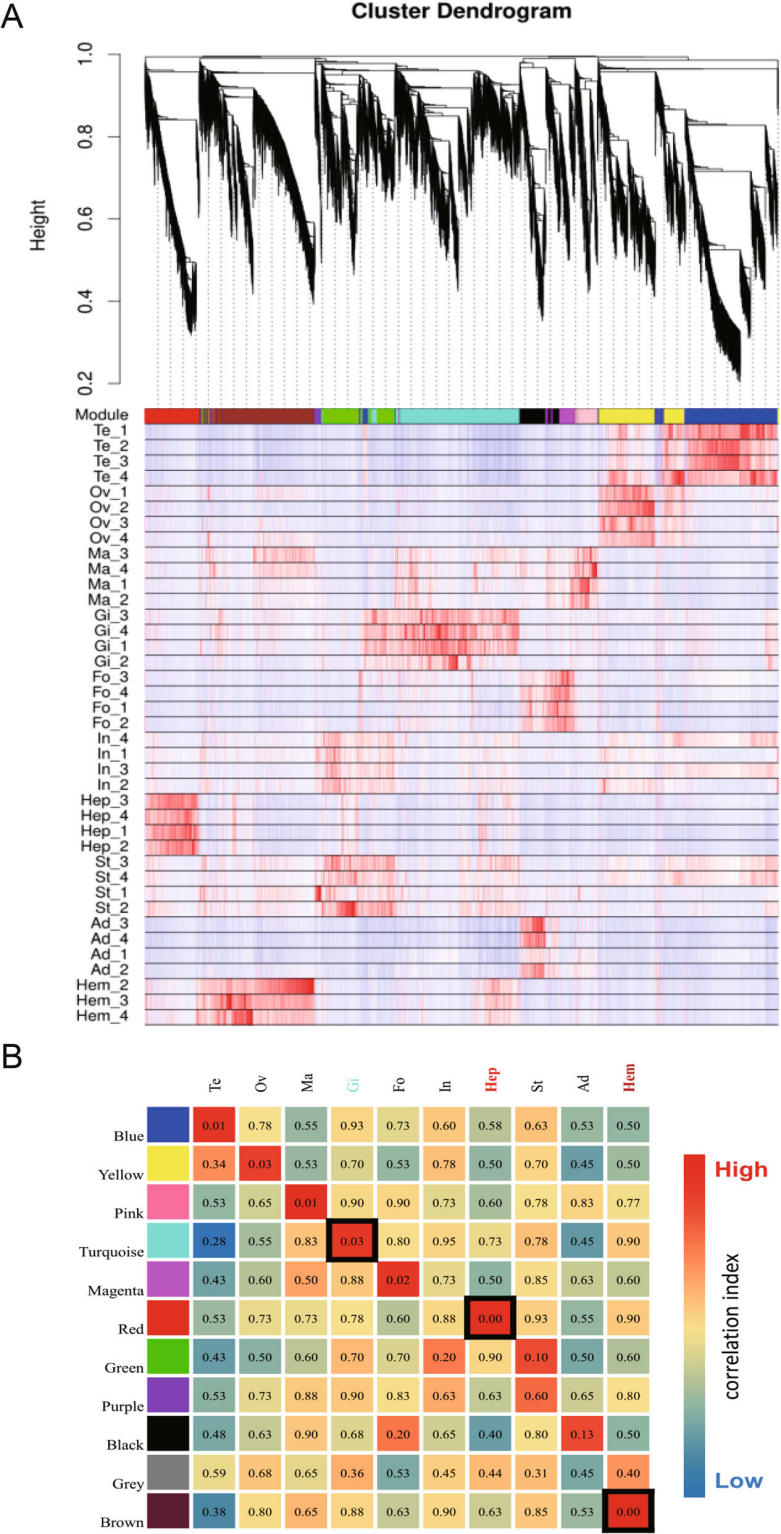
Co-expression network analysis of organ-specific genes in the hard clam, with focus on candidate immune response genes in hemolymph. **a** Module construction of gene-expression network for 39 samples. Te, testis; Ov, ovary; Ma, mantle; Gi, gill; Fo, foot; In, intestine; Hep, hepatopancreas; St, stomach; Ad, adductor; Hem, hemolymph. **b** Correlation matrix between modules and organs. *P* value is presented in each cell, and color indicates correlation coefficient

To explore roles of IAPs in innate immune response of the hard clam, we performed weighted gene co-expression network analysis (WGCNA) on 39 transcriptomes from 10 organs, focusing on genes showing increased connectivity in hemolymph, hepatopancreas, and gills, the primary organs in initial defense against pathogens. First, we identified 19,548 organ-associated genes that were differentially expressed between any two organs. Our analysis identified 11 gene modules with brown, red, and turquoise modules displaying significant correlation with hemolymph, hepatopancreas, and gills, respectively (Fig. 3a, b). The hard clam showed clear organ-specific transcriptomic signatures. Genes prominently expressed in hemolymph are associated with complement and coagulation cascades and the TNF signaling pathway, and these transcriptomic signatures were shared with other molluscs (Additional file 5: Fig. S3). Particularly, genes associated with apoptosis were extensively enriched in transcriptomes of hemolymph and gill (Additional file 5: Fig. S3 and Additional file 6: Fig. S4). In the co-expression network of top 20 KEGG pathways enriched in hemolymph specifically expressed genes (brown module genes), the apoptotic pathways were interconnected with NF-kappa B signaling, TNF signaling, NOD-like receptor signaling, and cancer pathways. These findings indicate that interactions among these pathways form a network that regulates cell death and survival, cellular homeostasis, and immunity in the hard clam (the brown shadow in Additional file 6: Fig. S4). In the top 20 enriched KEGG pathways of the brown module, many genes were annotated as IAPs and enriched in apoptotic pathways (29/45). IAPs also showed an extensive and robust co-expression with other genes in the brown module at weighted correlation coefficient cutoff of > 0.35 (top 3%) (Additional file 7: Fig. S5). This finding indicates that apoptosis regulation may play an important role in immune response in hard clam.

### IAP expansion by tandem duplication and retroposition

**Fig. 4:**
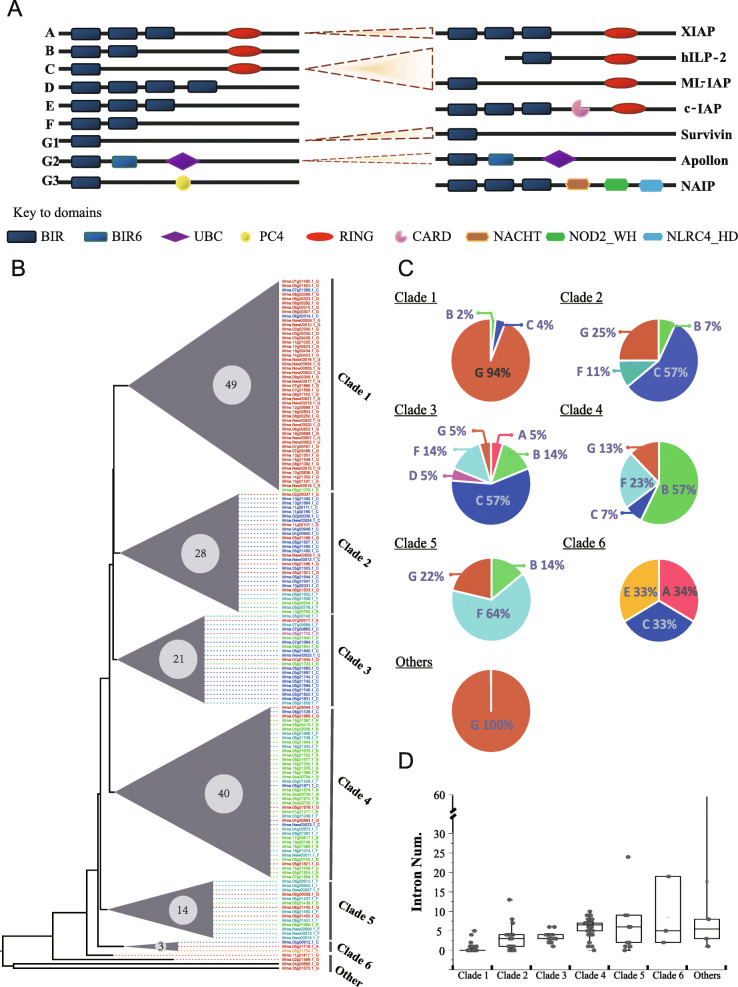
Domain architectures of IAPs from the hard clam (left, identified in this study) and humans (right, modified from Kocab et al. 2016) (**a**); phylogenetic tree of 159 IAPs of the hard clam (**b**); distribution of domain architectures (**c**) and intron number from different phylogenetic clades (**d**)

**Fig. 5:**
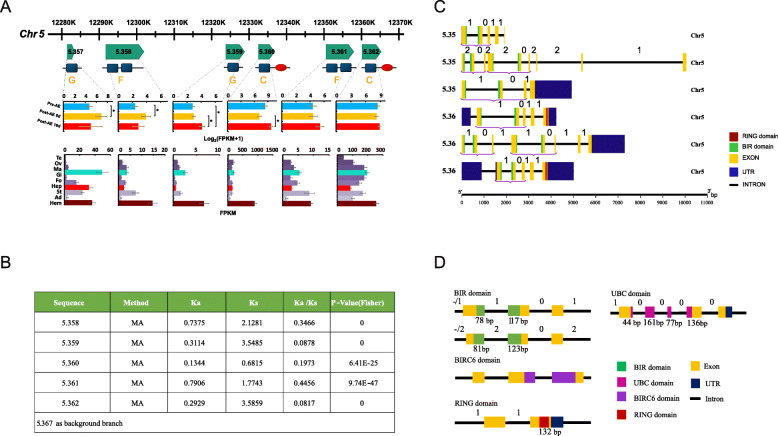
Divergence in expression profile of duplicated IAPs in the hard clam. **a** Tandem duplication of six IAPs in a ~ 90 kb region, with expression divergence among different organs and stages during aerial exposure. **b** Selective pressure on the six duplicated IAPs. **c** Gene model prediction for the six duplicated IAPs; lines represent introns, and boxes represent exons. Regions encoding different domains are colored accordingly. Numbers above introns indicate phase of each intron. **d** Boundaries of domains found in hard clam IAPs

Some of the BIR-domain-containing genes (Fig. 2) were incomplete, and further analysis identified 159 bona fide IAPs (Figs. 1a and 4b), giving the hard clam the largest IAP gene repertoire identified to date. The expansion of IAP family in the hard clam involved multiple events of local tandem duplication and retroposition. Massive tandem duplications were observed on chromosomes (Chr) 5, 6, and 7. Among the 159 IAPs, 59 (37.1%) were densely linked in tandem arrays on Chr 5, while 22 (13.8%) and 12 (7.6%) were tandemly duplicated on Chr 6 and 7, respectively (Fig. 1a). An example array contains 6 tandemly replicated IAPs located at 12,280–12,370 kilobase (kb) on Chr 5, transcribed in the same direction without other genes interspersed in between (Fig. 5a).

In addition to tandem duplication, a significant proportion of IAPs (51, 32.1%) were intronless and appeared to be randomly distributed across chromosomes (Additional file 8: Table S11), indicating retroposition. Phylogenetic analyses of the 159 IAPs revealed 6 discrete clades with small genetic distances and 4 other genes outside these clades. Clade 1 contained 49 IAPs (Fig. 4b), of which 42 were intronless and showed similar domain architectures (one exon encoding BIR with no intron), suggestive of a retroposition burst from a common “parental” gene. Thus, we speculate both tandem duplication and retroposition fueled the evolutionary expansion of IAPs in the genome of the hard clam.

Mechanistically, gene duplication can be facilitated by associated TEs. Analysis of TE distribution revealed that DNA transposons were significantly enriched (*p* < 0.01) around IAP genes (average density = 0.12) compared to other genes in the genome (average density = 0.05), while other types of TEs (including LINE, LTR, and SINE) showed no significant difference (Additional file 9: Fig. S6). This finding indicates DNA transposons may have played a role in the massive expansion of IAP genes in the hard clam genome.

### Frequent domain shuffling led to IAP structure diversity

To understand the evolution of IAPs after duplication, we classified the domain architecture of clam IAPs into seven types, A–G (Fig. 4a), based on copy number of BIR and RING domains, which are key to the function of IAPs. Extensive domain shuffling was found in hard clam IAPs. As revealed by domain architecture classification and phylogenetic analysis (Fig. 4b), IAPs that clustered together may be derived from the same ancestral gene, and domain architecture varied within the same cluster. Clam IAPs in the same clade (2–5) were comprised of 3 to 6 different domain architecture types, with each clade having one dominant domain architecture (> 50%) and varying proportions of derived types (36% to 43%) (Fig. 4c). The diverse domain structure is derived from domain shuffling, i.e., by gaining or loss of the BIR/RING domains. For example, the 6 tandemly duplicated IAPs in a 90-kb region on Chr 5 (Fig. 5a, Additional file 8: Table S11), displayed three types of domain architectures, C, F, and G (Fig. 4a), indicating domain shuffling has occurred during or after the tandem duplication.

To investigate how domain shuffling has affected *IAP* gene structure, we examined intron-exon structure of BIR, RING, BIRC6, and UBA domains (Fig. 5d). Remarkably, each domain displayed highly conserved intron-exon structures. While the RING domain was bound within a single exon at the end of the gene, most hard clam IAPs (except for those originating from retroposition) had an initial BIR domain that spanned the first two exons. The first two BIR-encoding exons were always followed by another exon flanked by phase 0 and phase 1 (or 2) introns (bracketed in Fig. 5c, d). This finding suggests that a BIR domain together with the successive exon may be inserted into an IAP as a multi-exon module, or similar flanking and internal intron phases allowed the domains to be “mixed and matched” from a pool of single-exon building blocks.

To determine if duplicated IAPs experienced different selection pressure, we calculated the nonsynonymous to synonymous substitution ratio (Ka/Ks) for the 6 tandemly duplicated IAPs on Chr 5 (Fig. 5a). The Ka/Ks of all 6 duplicated IAPs was significantly less than 1 (*p* values range from 0~9.74E−47, Fig. 5b), indicating that they were functionally constrained by purifying selection. The variation of Ka/Ks from 0.0817 to 0.4456 suggests that the duplicated genes had experienced different levels of purifying selection, which could be important for sequence and functional divergence.

### Functional divergence of duplicated IAPs

**Fig. 6:**
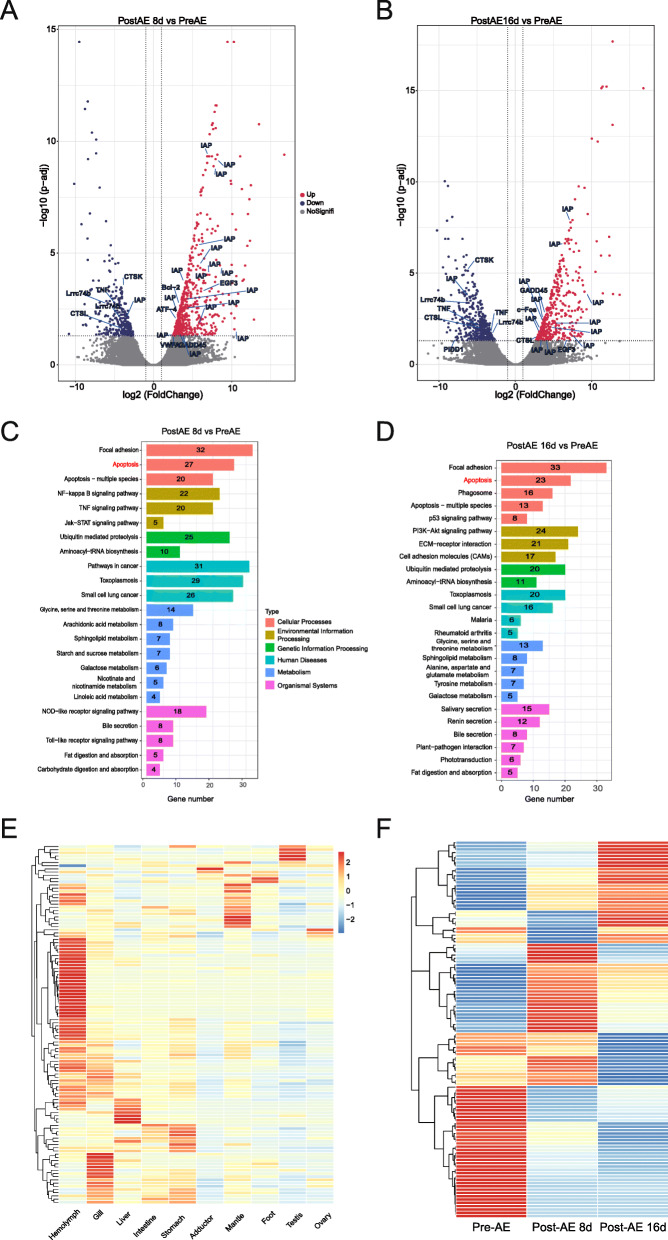
Transcriptome response of hard clams subjected to air-exposure stress and IAP expression divergence. **a**, **b** Volcano plots of differential expressed genes (DEGs) (8 days vs 0 days and 16 days vs 0 days). **c**, **d**, KEGG pathways enriched in DEGs (8 days vs 0 days and 16 days vs 0 days). **e**, **f** Coordinated expression of hard clam IAPs among organs and during aerial exposure. IAPs with average FPKM< 0.1 were considered silent and excluded

To understand how IAPs respond to environmental stressors, we performed RNA-seq on days 0, 8, and 16 after adult clams were subjected to aerial exposure. There were 632 genes upregulated and 343 downregulated on day 8, while 506 upregulated and 532 downregulated on day 16. Notably, the apoptotic pathway was one of the most significantly enriched pathways in transcriptomes of clams subjected to an 8- or 16-day aerial exposure (Fig. 6c, d). This indicates that apoptosis was functionally important for maintaining homeostasis as aerial exposure causes decreases in pH and oxygen levels, leading to dysfunction in cellular homeostasis. The enrichment in apoptosis-related genes was mainly attributed to the expression of the IAP family: 17 of the 27 differentially expressed genes (DEGs) from the apoptotic pathway were IAPs on day 8, and 12 out of 23 on day 16 (Fig. 6a, b). Besides aerial exposure, we also compared transcriptomes of hard clams subjected to high temperature, low oxygen, and low salinity. In all, 134 IAPs were differentially expressed under at least one environmental stressor, and among 27 DEGs shared by all stressors, three were IAPs (Additional file 10: Fig. S7). These findings indicate that over 84% of the expanded IAPs (134 of 159) are involved in stress response in the hard clam with remarkable specificity, which highlights the importance of the expansion and diversification of IAPs in stress response and adaptation.

The duplicated IAPs exhibited diverse expression profiles, both spatially in different organs and temporally during environmental stress, suggesting functional divergence and coordination. Functional divergence is implied not only by the dedicated organ-specific expression of different IAPs (Fig. 6c), but also by differential response or expression pattern of IAP members to specific environmental stressors, exemplified by the differentiated responsiveness to aerial exposure (Fig. 6d), and divergent sensitivity or responsiveness to different environmental stressors (Additional file 11: Fig. S8), where the hemolymph-expressed IAPs (brown module of WGCNA) showed clear expression divergence in response to different stressors. The IAPs in green clade are sensitive to aerial exposure stress, while that in purple clade are sensitive to low salinity, and that in blue clade are sensitive to heat stress.

Both at the clade level and regarding to the structural type (Additional file 12: Fig. S9 and Additional file 13: Fig. S10), the expression of IAPs in response to environmental stress was highly variable. Generally, IAPs in clades 2, 3, and 5 displayed similar expression dynamics in that they were all upregulated when clams faced thermal, osmotic, or aerial exposure stress, but with different expression levels and organ specificity. Compared to IAPs in clades 2, 3, and 5, those in clade 4 were less sensitive to environmental variables and clade 6 showed nearly opposite expression patterns. IAPs with different structural types (A~E) also displayed divergent expression patterns, for example, D-type IAPs which were particularly sensitive to environmental stressors exhibited clear organ specificity: high expression in the mantle and low expression in the hemolymph. In contrast, B-type IAPs showed similar response to environmental stress but had the highest expression in the hemolymph. E-type IAPs showed an opposite trend to B and D types in responding to multiple stressors.

The notion of orchestrated expression after duplication is reinforced by the six tandemly duplicated IAPs on Chr 5 (Fig. 5a), which showed constitutive responsiveness to aerial exposure and divergent organ expression bias: genes 5.357 and 3.578 reached the highest expression levels at 8 days post aerial exposure, 5.359 and 3.360 were highly expressed at 16 days, and 3.61 and 3.62 did not exhibit significant changes. With respect to differential expression among organs, 5.357 showed the highest expression in gills, 5.357~5.360 were prominently overexpressed in hemolymph, while 5.361 and 5.362 were uniformly expressed.

### Evolution of IAP repertoires among multiple phyla

**Fig. 7:**
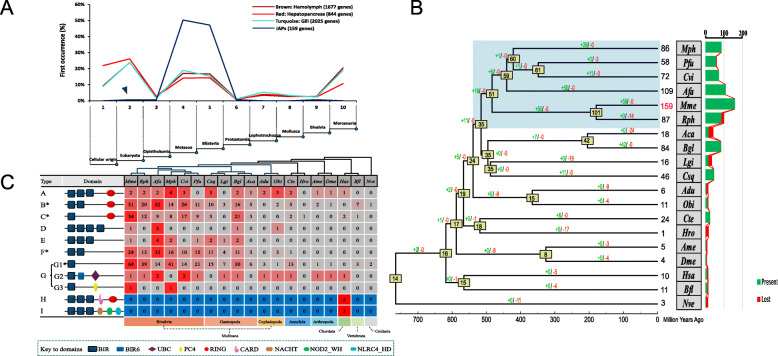
Origin of hard clam IAPs and IAP evolution in different metazoan lineages. **a** Evolutionary origin of IAPs and organ-specific genes in hard clam. Phylostratigraphic analyses of IAPs (blue), hemolymph-specific (brown), hepatopancreas-specific (red), and gill-specific (turquoise) genes across 10 phylostrata. Only genes for which at least one significant BLAST hit (< e−10) was returned were included. **b** Expansion and contraction of IAP repertoire across 18 species from different metazoan lineages. Gene gain and loss events are mapped to the species tree, indicated by green and red numbers, respectively. Numbers in yellow boxes indicate the predicted ancestral IAP number of each node. The blue block indicates Bivalvia. Mph, *Modiolus philippinarum*; Pfu, *Pinctada fucata*; Cvi, *Crassostrea virginica*; Afa, *Azumapecten farreri*; Mme, *Mercenaria mercenaria*; Rph, *Ruditapes philippinarum*; Aca, *Aplysia californica*; Bgl, *Biomphalaria glabrata*; Lgi, *Lottia gigantea*; Csq, *Chrysomallon squamiferum*; Adu, *Architeuthis dux*; Obi, *Octopus bimaculoides*; Cte, *Capitella teleta*; Hro, *Helobdella robusta*; Ame, *Apis mellifera*; Dme, *Drosophila melanogaster*; Hsa, *Homo sapiens*; Bfl, *Branchiostoma floridae*; Nve, *Nematostella vectensis.* **c** Distribution of IAPs with different domain architectures in 19 species of Metazoa. Asterisk indicates domain architectures expanded in bivalves with a significant corrected *P* value (*P* < 0.0001) from Chi-square tests for overrepresentation, using all annotated genes as background

To explore the evolutionary origin of extant IAPs, a phylostratigraphic approach with an E < e^−10^ cutoff was applied to estimate the ages of IAPs and organ-specific genes. We defined 10 phylogenetic ranks (phylostrata) based on the NCBI taxonomy database and using the first phylostratum (PS1) as the point of origin of cellular life (i.e., oldest genes), and the last phylostratum (PS10) as hard clam lineage under investigation (i.e., youngest genes). Organ-specific genes in the hemolymph, hepatopancreas, and gill showed a similar gene-age pattern, in which only hepatopancreas-specific genes contained an increased percentage of oldest (+ 11%) and decreased percentage of youngest (− 9%) genes. Most expanded IAPs of the hard clam pre-date the origin of Metazoan (50%) and Bilateria (47%), while one IAP (ID, Mme.02 g01599.1, indicated by a blue triangle in Fig. 7a) first appeared with the emergence of eukaryotes. This gene was unique in structure and size at a length of 90.83 kb (Additional file 8: Table S11) and contained a BIR6 domain (G2 type in Fig. 4a). Similarly, a mammalian IAP, termed Apollon (4.88 kb and also containing a BIR6 domain), structurally differs from classical metazoan-specific IAPs and shows homology to a protein in yeast [33]. Our results support the existence of two lineages of IAPs, one is the BIR6-containing IAPs which is ancient and originated in eukaryotes, and the other lineage is the BIR-containing IAPs which are shared by multicellular organisms [33].

**Fig. 8:**
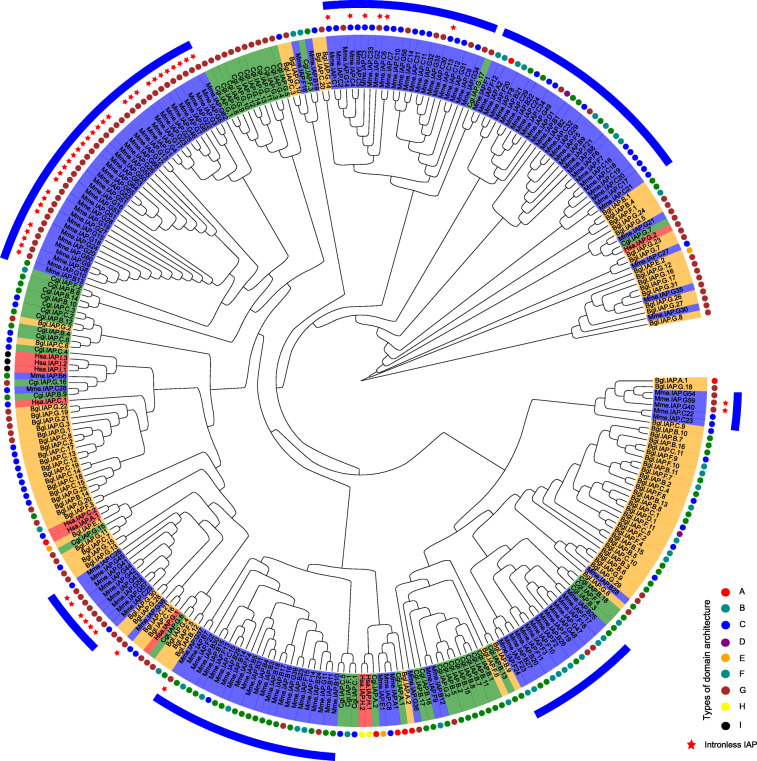
Phylogenetic analysis of IAPs from *Mercenaria mercenaria* (Mme), *Crassostrea virginica* (Cvi)*, Crassostrea gigas* (Cgi), *Biomphalaria glabrata* (Bgl), and *Homo sapiens* (Hsa) Dendrogram was generated using Bayesian analysis with WAG substitution model. Domain architecture is defined in Fig. 4a

We further identified intact IAPs among 19 species and used a phylogenetic framework to trace the evolutionary fates of these IAPs across multiple animal phyla (Fig. 7b). The most recent common ancestor (MRCA) of all metazoans was estimated to have had 13 IAPs, while the successive expansion of the IAP family likely occurred in the common molluscan ancestor and subsequent lineages. There was a significant expansion in bivalves that did not occur in other taxa (blue block in Fig. 7b). The expansion of bivalve IAPs was mainly lineage-specific (Fig. 8), as paralogs within the same species clustered together, suggesting that their expansion occurred after speciation. The significant expansion of IAPs in multiple bivalve lineages implies convergent evolution of enhanced apoptosis regulation in stationary bivalves who have to cope with environmental stress without the capability of avoidance.

The massively expanded bivalve IAPs were dominated by B, C, F, and G1 domain structural types (Fig. 7c), which consist of one or two BIR domains, coupled or uncoupled with a RING domain. They differed considerably from other IAP types (i.e., A, D, E, G2, and G3), which have fewer copies (< 5) in all animal phyla. Additionally, the domain arrangement of D, E, and G3 types may have occurred via domain gain (an extra BIR), loss (missing RING domain), and co-option (co-opted PC4), while H and I types in humans are likely structural innovations that co-opted the CARD, NACHT, NOD2_WH, and NLRC4_HD domains.

## Discussion

In multicellular organisms, development and homeostasis require a delicate balance between pro- and anti-apoptotic machineries. However, little is known about the evolution of these systems [10]. To understand apoptosis regulation in hard clam, and more generally the evolution of apoptosis-related genes, we produced a chromosome-level assembly of the hard clam genome and compared it with other genomes with a focus on IAPs. We surveyed IAPs across molluscan and non-molluscan genomes and characterized their domain organization. This allowed us to examine the evolution of putative anti-apoptotic machineries across multiple phyla. Our results indicate that, while the IAP family emerged early in metazoan evolution, it underwent massive lineage-specific expansions in several bivalve molluscs, followed by domain shuffling and functional diversification. The expansion, powered by tandem duplication and retroposition, led to the extant IAP repertoire, which may have played a key role in the evolution of bivalves particularly in adaptation to harsh environmental conditions.

### Evolution of sophisticated apoptosis system in bivalves

Mollusca is the second-largest phylum of metazoans with diverse species found in marine, freshwater, and terrestrial environments. Bivalve molluscs are mostly stationary filter-feeders with many living in intertidal zones or shallow waters where environmental conditions fluctuate widely. Therefore, cells in bivalves must constantly monitor and respond to their environment. Cells exposed to a wide range of potentially apoptotic signals must avoid continuously triggering premature or unnecessary apoptosis. For this reason, bivalve cells may need particularly strong anti-apoptotic systems [10, 23, 34–36]. Our analyses reveal that the hard clam and other bivalve genomes contain a great expansion of domains commonly associated with genes from apoptosis pathways (Fig. 2). The expansion and divergence of these genes encoding anti-apoptotic signals may have contributed to the complex and sophisticated apoptosis system in bivalves and to their robust ability to tolerate stress [23, 34].

More specifically, the IAP family is greatly expanded in stem-molluscs, stem-bivalves, and sub-lineages (Fig. 7b), and their subsequent divergence in architecture and expression indicates that they underwent many cycles of expansion, genetic innovation, and adaptive evolution. The fact that IAPs continued their expansion in several different bivalve lineages suggests convergent evolution, and their expansion is likely critical for the adaptation of bivalves. In the hard clam, a large proportion of expanded IAPs were highly expressed in the hemolymph (Figs. 3, 5a, and 6e), highlighting their potential roles in immune response. Transcriptomic profiling of different organs and hard clams under various environmental stressors revealed that the majority (84%) of the expanded IAPs are involved in stress response with orchestrated expression and remarkable specificity in organ distribution and response to different stressors. The great expansion and functional divergence of IAPs may be partly responsible for the enhanced environmental resilience of hard clams and other bivalves. As IAPs are important for immune response, the great expansion of IAPs may also explain why the hard clam has fewer pathological conditions [27]. The expression of IAPs is highly upregulated following immune challenges in the carpet shell clam *Ruditapes decussatus* [37] and in the Pacific oyster *Crassostrea gigas* especially in response to viral infection [38, 39], highlighting the importance of IAPs in bivalve immune response. As benthic filter-feeders, bivalves face strong challenges from diverse pathogens, and reinforcing their innate immune system is critical to their survival [34]. Thus, the expansion and diversification of the IAP gene family in different bivalve lineages may be driven by convergent evolution of enhanced apoptosis in response to heightened biotic and abiotic stress. It should be noted that not all bivalves living in stressful environments, and there is no perfect correlation between the current habitat and the number of IAPs. The expansion of IAPs in molluscs may also be driven by lineage-specific retroposition mediated by TEs or other mechanisms not yet known.

### Novel orchestration of IAP gene family after duplication and co-option

Gene duplication is a major source of evolutionary novelty [40–42]. Duplicated genes provide the raw material for the evolution of new genes and new functions. Segmental and tandem duplication, and transposition (primarily retroposition) [43], represent three principal evolutionary processes for gene duplication [42]. Tandem duplication is responsible for the expansion of several multigene families in bivalves, including HSP70s, nAChRs (nicotinic acetylcholine receptors), TLRs, C1qDC, and TNFRs [23, 39, 44]. Our results indicate that the expansion of IAPs was driven not only by tandem duplication, but also by retroposition, which has also been reported for the expansion of nAChRs in the pearl oyster [44]. Most IAPs duplicated by retroposition were not transcribed (possibly due to lack of promoters), while a small portion of these IAPs were integrated with *cis*-acting elements and displayed high levels of expression. Retroposition-derived duplicates were silent with respect to domain reforming (96% remained in G1 type), while other duplicates had undergone active domain shuffling (27~50% duplicates in clade 2~6 had variant domain architectures that differed from the dominant types) (Fig. 4). The frequent occurrence of B, C, F, and G types in clade 2~5 suggests that these are quite inter-transformable via domain co-option, or gaining or loss of BIR and RING domains. This interpretation is further supported by the high level of structural constraint observed in hard clam IAPs, where their domains conform to precise boundaries within their encoding exons. These boundaries may be remnants of an ancestral domain-module structure [45]. The conservative intron phases of RING and BIR domains allow IAPs to go through frequent shuffling.

While silent IAPs (expressed at FPKM < 0.1) were disregarded in our analysis, duplicated IAPs in the hard clam showed an intriguing arrangement of expression patterns (Fig. 6e, f), indicating functional divergence with respect to gene regulatory networks in different organs and in response to aerial exposure. Both at the clade level or on the basis of the structural type (Additional file 12: Fig. S9 and Additional file 13: Fig. S10), IAPs showed orchestrated expression, in terms of tissue expression specificity and responsiveness to environmental stress. It is interesting to note that the six duplicated IAPs on Chr 5 exhibited variations in domain architecture and transcriptional profile, while all were subjected to purifying selection. The paradox of constraints from purifying selection and their fast structural and expression divergence can be explained by a two-stage evolutionary model of gene duplication based on analyses of recent duplications observed in bacterial, archaeal, and eukaryotic genomes [46]. In the first stage (the early phase of evolution), the function of duplicated genes is retained through purifying selection and the short-term advantage of protein dosage effect, and in the later stage of evolution, gene duplications provide a long-term advantage by giving rise to new functions. The evolution of hard clam IAP duplicates appears to span both stages, and as a result, novel orchestration of IAP expression has evolved. Here, novel orchestration refers to the incorporation of different GRNs (gene regulation networks) or changes in the expression levels of duplicated IAPs.

The patterns of transcriptional divergence underline the contribution of gene duplication and co-option to evolutionary adaptations and are, in general, compatible with results reported for pathogen-associated pattern recognition receptors (PAMRs) in oysters [39] and fibrinogen-related proteins (FREPs) in the freshwater snail [47]. Gene co-option here refers to divergence in expression of duplicated genes that implies some functional difference. In addition to divergence in expression profile, the divergence in domain structures may also have functional implications. Although our results indicate that gene co-option and selection pressure may underpin the orchestration of IAPs in the hard clam, much remains to be learned about the precise evolutionary processes that led to the incorporation of the extant IAP repertoire into different gene regulatory networks, and detailed differentiation of molecular function of extant IAPs.

### Origin and evolutionary dynamics of the IAP gene family

Previous studies in mammals including humans, as well as those in insects, nematodes, and yeast, have categorized IAPs into two groups [33]. The first group consists of “real” IAPs, which contain between 1 and 3 BIR domains and often display a RING-finger domain; members of this group inhibit cell death, either via deactivation of caspase or via signal transduction to activate NF-kB-mediated inhibition of apoptosis. These IAPs have been identified in multicellular organisms from *Drosophila* to mammals, but are not present in plants, yeast (possibly lost during evolution), protozoans, or *C. elegans* [33]. The second group of IAPs (containing BIR6 domain), found in a wider range of organisms, including yeast, nematodes, insects, and mammals, are evolutionarily ancient than those of the first group and are involved in mitosis and cytokinesis rather than in inhibition of apoptosis [33, 48]. The IAPs in this second group differ from those in the first group in terms of the function as well as the structure of the BIR domains [33]. IAPs in the second group correspond to the G2 type of the hard clam (Fig. 7c), which contains a rare BIR6 domain, and has domain arrangement similar to human Apollon, also an IAP of the second group. The G2 type gene (Mme.02 g01599.1), which has only one copy and appears to be conserved among the species surveyed in this study (Fig. 7c), was predicted to have originated in eukaryotes (Fig. 7a). This gene is extremely long (90.83 kbp); contains 59 introns, significantly more than other IAPs; and exhibits decreased sequence similarity (Fig. 4b). Although hard-clam IAPs can also be divided into the same two groups, the vast expansion was mainly found in the first group. This is clear from the fact that the expanded IAPs largely belong to the B, C, F, and G1 types (Fig. 7c) and originated in Metazoa and Bilateria (Fig. 7a).

Although our findings show that the expansion of clam and bivalve IAPs are principally attributed to the duplication of B, C, F, and G1 types (Fig. 7c), different BIRs may have diverged in function. For instance, the mammalian XIAP gene, which has 3 BIR domains and a RING domain, contains a conserved surface groove in the second and third BIR domains (2nd-BIR, 3rd-BIR), which can interact with caspase 3/7 and caspase 9, respectively; importantly, the first BIR domain (1st-BIR) does not carry such an interacting site [19, 33, 49, 50]. A similar phenomenon exists in bivalves. In oysters, the second, but not the first, BIR domain of CgIAP2 can interact with caspase-2 [14]. Functional differences among the BIR domains of different types remain unknown, especially because IAPs of one type may have evolved from different parental IAPs. Therefore, due to the complexity of the bivalve IAP repertoire, further studies are needed to understand their functions. Additionally, the D (containing four BIRs) and E (containing three BIRs without a RING domain) type IAPs are molluscan novelties. Unlike the B, C, F, and G1 types, which are highly expanded and can be interchanged via domain gain or loss, the D and E types are likely under copy-number constraints (< 3 copies across Mollusca). The G3 type (containing a BIR and PC4) is an evolutionary innovation of the hard clam and mussels. All these factors contribute to the complexity of IAPs observed in bivalves.

### Hallmarks of constraint on the modular exon structure

The domains of the clam IAPs possess constrained modular exon structures. Each domain type displays remarkably conserved exon distribution across all clam IAPs, with the exception of those duplicated by retroposition (Fig. 5c, d). Most BIR domains are encoded by two exons, which, on average, span the last ~ 80 base pairs (bp) of exon 1 and the first ~ 120 bp of exon 2. This pattern is consistent with the second group of human IAPs (including the ancient IAP Apollon), in which the BIR domain is encoded by two exons, different from XIAP and cIAPs [33, 51]. In XIAP and cIAP, BIR1 and BIR2 domains, as well as half of the BIR3 domain, are encoded by exon 1; the rest of the BIR3 domain is encoded by exons 2 and 3. This indicates that BIR domains in hard clam succeeded in constraining ancient IAP exon structures, while mammalian XIAP and cIAPs possibly evolved via exon shuffling. Despite such striking dissimilarities in intron-exon structures of these BIRs, the RING domain, which is encoded by the last exon in both clams and mammals, exhibits conservation across species.

## Conclusions

The IAP family is involved in critical signaling pathways mediating cell death and survival. Currently, little is known about the evolution of IAPs in Mollusca, the second largest phylum in the animal kingdom. In this study, we produced and characterized a chromosome-level assembly of the hard clam genome with a focus on the diversity, characteristics, and evolutionary history of molluscan IAPs. The hard clam genome is highly polymorphic and encodes a large gene repertoire that is enriched for immune and stress response genes. Our analyses demonstrate that the hard clam genome possesses the largest IAP family among all available metazoan genomes, with far more IAPs than that found in humans and model organisms such as nematodes, fruit flies, and mice. The dramatic expansion of IAPs in the hard clam was driven by both tandem duplication and retroposition, with more than one third of expanded IAPs located on one chromosome. The massive expansion of IAPs was lineage-specific and occurred in multiple molluscan lineages, especially stationary bivalves, indicative of convergent evolution of sophisticated apoptosis regulation in response to environmental stress. Reconstruction of IAP gene family evolution indicated that duplicated IAPs were subjected to frequent domain shuffling that shaped functional divergence. Transcriptomic analyses of IAP expression induced by multiple stressors, such as aerial exposure, low salinity, heat, hypoxia, and pathogens, suggest that most expanded IAPs (84%) are actively involved stress response which may underpin the remarkable environmental resilience of hard clams and other bivalves. As additional annotated molluscan genome assemblies become available, functional analysis on molluscan IAPs will enable the precise delineation of the evolutionary processes that drove the expansion of the IAP family. Future studies may uncover how these duplicated IAPs were co-opted into GRNs to function in environmental resilience.

## Methods

### Sample preparation and sequencing

Genomic DNA of an adult hard clam *Mercenaria mercenaria* (collected from Qingdao, Shandong, China) was extracted from the adductor muscle for whole genome sequencing, using a QIAGEN DNeasy Kit (QIAGEN, Shanghai, China). A paired-end Illumina sequence library with insert size of 350 bp and a 10x Genomics linked-read library were constructed and sequenced with Illumina HiSeq X. A PacBio library was constructed and sequenced with a PacBio Sequel platform. Low-quality reads and sequencing-adaptor-contaminated reads were removed. Finally, a total of 986.55 GB clean data were used to assemble the *M. mercenaria* genome. RNA isolation and construction of RNA-seq libraries for different organs (foot, adductor, visceral mass, gonad, mantle, and gill) from the same *M. mercenaria* individual were carried out per Song (2016) [52] and sequenced with Illumina HiSeq X, per the manufacturer’s instructions. Following quality control, clean reads were assembled using Trinity, and prepared for genome annotation.

### Size, assembly, and evaluation of the hard clam genome

Jellyfish (v2.0) [53] was used to estimate genome size based on *k*-mer distribution using high-quality reads from short-insert size libraries.

Using long reads generated by the PacBio Sequel platform, contigs were assembled using the WTDBG software v2.2 (https://github.com/ruanjue/wtdbg) with the following parameters: “--node-drop 0.20 --node-len 2304 --node-max 500 -s 0.05 -e 3”. This assembly was then polished using Quiver (smrtlink 6.0.1) with default parameters [54]. Heterozygosity in the assembly was removed via Purge Haplotigs software (v1.0.4) [55]. The resulting contigs were connected to super-scaffolds using 10x Genomics linked-read data and fragScaff software (v140324) with the following parameter settings: “-maxCore 200 -m 3000 -q 30 -C 5” [56]. Conversely, short Illumina reads were used to correct any remaining errors by pilon (v1.22) with parameters set as follows: “-Xmx300G --diploid --threads 20” [57]. Finally, Hi-C data were used to generate the chromosomal-level assembly of *M. mercenaria* genome with Lachesis software (v201701) with default parameters. After that, chromosomes were numbered by Lachesis without sorting in descending size order.

To evaluate the accuracy of the assembly, short Illumina reads were mapped to the *M. mercenaria* genome using BWA (v 0.7.8-r455) with parameter settings at: “-o 1 -i 15” [58]. Variant calling was performed with SAMTOOLS (SAMTOOLS, RRID:SCR 002105) [59]. Assembly completeness was assessed based on universal single-copy orthologs (BUSCO) (BUSCO, RRID:SCR 015008) [60] by searching against metazoan BUSCO (v4.0.1) [61].

### Genome annotation

Homologous comparison and de novo prediction were employed to annotate the repetive sequences in the *M. mercenaria* genome. RepeatMasker and the associated RepeatProteinMask (RepeatMasker, RRID:SCR 012954) [62] were used for homologous comparison to align against the Repbase database [63]. For ab initio prediction, LTR_FINDER (LTR_FINDER, RRID:SCR 015247) [64], RepeatScout (RepeatScout, RRID:SCR 014653) [65], and RepeatModeler (RepeatModeler, RRID:SCR_015027) (v2.1) were used to construct a de novo candidate database of repetitive elements. Using this database, repeated sequences were then annotated using RepeatMasker. Tandem repeat sequences were predicted de novo using TRF (v 4.07b) [66].

Genes were annotated using a combination of homology-based prediction, de novo prediction, and transcriptome-based prediction. For homologous annotation, protein sequences from other molluscs, including mussel *Bathymodiolus platifrons* (Bpl, https://datadryad.org/stash/dataset/doi:10.5061/dryad.h9942), mussel *Modiolus philippinarum* (Mph, https://datadryad.org/stash/dataset/doi:10.5061/dryad.h9942), scallop *Mizuhopecten yessoensis* (Mye, GCF_002113885.1_ASM211388v2), scallop *Azumapecten farreri* (Afa, http://mgb.ouc.edu.cn/cfbase/html/download.php), pearl oyster *Pinctada fucata* (Pfu, http://gigadb.org/dataset/100240), Eastern oyster *Crassostrea virginica* (Cvi, GCF_002022765.2_C_virginica-3.0), apple snail *Pomacea canaliculata* (Pca, GCF_003073045.1), limpet *Lottia gigantea* (Lgi, GCF_000327385.1_Helro1), Octopus *Octopus bimaculoides* (Obi, GCF_001194135.1_Octopus_bimaculoides_v2_0), and lancelet *Branchiostoma floridae* (Bfl, GCF_000003815.1_Version_2), were aligned against *M. mercenaria* genome using TBLASTN (TBLASTN, RRID:SCR 011822) [67]. Hits generated using the Basic Local Alignment Search Tool (BLAST) were then conjoined via Solar software (v 0.9.6) [68]. GeneWise (GeneWise, RRID:SCR 015054) [69] was used to predict the exact gene structure of the corresponding genomic region on each BLAST hit. Homology predictions were denoted as “homology-set.” Approximately 50.4 GB high-quality RNA-seq data were assembled via Trinity (v2.0) [70], and the assembled sequences were aligned against the *M. mercenaria* genome to assemble spliced alignment [71]. Using PASA (v2.0.2), effective alignments were clustered based on genome-mapping location and assembled into gene structures. Gene models created via PASA were denoted as PASA Trinity set (PASA-T-set). We simultaneously used five tools in Augustus (Augustus, RRID:SCR 008417) [72], GeneID (v1.4) [73], GeneScan [74], GlimmerHMM (GlimmerHMM, RRID:SCR 002654) [75], and SNAP (v 2013-02-16) [76] for ab initio prediction, in which Augustus, SNAP, and GlimmerHMM were trained using PASA-H-set gene models. In addition, RNA-seq reads were directly mapped to the *M. mercenaria* genome using Tophat (Tophat, RRID:SCR 013035) [77]. Mapped reads were assembled into gene models (Cufflinks-set) using Cufflinks (Cufflinks, RRID:SCR 014597) [78]. All gene models were integrated via EvidenceModeler (EVM) [71]. Weights for each type of evidence were set as follows: PASA-T-set > Homology-set > Cufflinks-set > Augustus > GeneID = SNAP = GlimmerHMM = GeneScan. To detect untranslated regions (UTRs) and alternate splicing variation, PASA2 was used to update the *M. mercenaria* genome. To achieve functional annotation, predicted protein sequences were aligned against public databases including SwissProt [79], NR database (from NCBI), InterPro [80], and KEGG pathway [81]. Of these, the InterproScan tool [82] and the InterPro database were used to predict protein function based on conserved protein domains and functional sites. KEGG pathway and SwissProt databases were used as the main source for mapping and identifying the best match for each gene.

### Phylogenetic reconstruction and divergence estimation

To ensure the representativeness and reliability of phylostratigraphic tree, we included 11 species—whose genomes are currently available—from each representative family in molluscs (Mytilidae, Pteriidae, Ostreidae, Pectinidae, and Veneridae for bivalves; Aplysiidae, Planorbidae, Lottiidae, and Peltospiridae for Gastropods; Architeuthidae and Octopodidae for Cephalopods) and 7 species from other phyla for downstream analysis. Hence, the nucleotide and protein sequences of those 18 species (*P. fucata*; *C. virginica; M. philippinarum*; *A. farreri*; *Ruditapes philippinarum* (Rph) [83]; *Chrysomallon squamiferum* (Csq): GCA_012295275.1; *L. gigantea*; *Biomphalaria glabrata* (Bgl)*:* GCA_000457365.1 ASM45736v1; *Aplysia californica* (Aca): GCF_000002075.1; *Architeuthis dux* (Adu) [84]; *O. bimaculoides*; *Capitella teleta* (Cte): GCA_000328365.1 Capca1; *Helobdella robusta* (Hro): GCA_000326865.1; *Apis mellifera* (Ame): GCF_003254395.2_Amel_HAv3.1; *Drosophila melanogaster* (Dme): GCF_000001215.4_Release_6_plus_ISO1_MT; *Homo sapiens* (Hsa): GCF_000001405.38_GRCh38.p12; *B. floridae; Nematostella vectensis* (Nve): GCA_000209225.1 ASM20922v1) were downloaded from public databases (see also database IDs above). The longest transcript was selected from alternate splice transcripts for each gene, and genes with ≤ 30 amino acids were removed. Gene families were constructed according to OrthoMCL pipeline using the parameter of “-inflation 1.5” (OrthoMCL, RRID:SCR 007839) [85].

The protein-coding sequences of single-copy genes were aligned using MUSCLE tool at default parameters [86]. Maximum-likelihood (ML) algorithm in RAxML software (v 8.0.19) with PROTGAMMAAUTO model [87] was used to analyze the phylogenetic relationships of *M. mercenaria*. Next, the MCMCtree program from the PAML package [88] was used to estimate the divergence time in the following manner: main parameter burn-in = 100,000, sample-number = 100,000, and sample-frequency = 2. The following time constraints were used to calibrate the phylogenetic tree: Bfl-Has (522.9 ~ 583.9 Mya); Mph-Afa (355.8 ~ 473.3 Mya); Lgi-Afa (510.9 ~ 519.7Mya); Obi-Afa (531.8 ~ 547.8 Mya); Nve-Bfl (625.5 ~ 973.7 Mya); Ame-Dme (291.3 ~ 358.9 Mya) from timetree; minimum 532 Mya and soft maximum 549 Mya, for the first appearance of molluscs [24]; minimum 550.25 Mya and soft maximum 636.1 Mya, for the first appearance of Lophotrochozoa [89].

### Gene family evolution and domain analysis

Evolutionary dynamics (expansion/contraction) of gene families were analyzed using CAFÉ (v.2.1) [90] with a stochastic birth and death model. Global parameter, λ, was estimated based on the phylogenetic tree and datasets of gene family clustering, which represented the birth and death rates of all gene families and identified significantly changed families (*p* < 0.05; Viterbi method in CAFÉ). Enrichment analyses of pathways and Gene Ontology (GO) terms were performed via EnrichPipeline [91] at *p* < 0.05. We then used the hidden Markov model (HMM) to search the main functional domains related to apoptosis in 19 metazoan species [24] based on the Pfam database. Next, the number of genes with apoptosis-related domains was counted (a domain with multiple copies in a protein was counted once). Chi-square tests were performed to assess overrepresentation in the *M. mercenaria* genome using all annotated genes in each species as background [23].

### Transcriptome profiling and gene co-expression network analysis of different organs

Ten adult organs (testis, ovary, mantle, gill, foot, intestine, liver, stomach, adductor, and hemolymph) were dissected from clams of the same cohort, with *n* = 3 for hemolymph and *n* = 4 for other tissues/organs. RNA was extracted from these 39 samples using a previously described protocol [52]. RNA-seq libraries were constructed using the NEBNext mRNA Library Prep Master Mix Set, as per the manufacturer’s instructions, and subjected to Illumina HiSeq X sequencing. High-quality RNA-seq reads were mapped onto the reference genome of *M. mercenaria* using Hisat2 (v2.0.4) [92]. HTseq [93] was used to calculate read count, and finally, gene expression levels in terms of FPKM were estimated according to the formula “FPKM = (number of reads in gene × 10^9^)/(number of all reads in genes × the gene length).”Differentially expressed genes (DEGs) were defined using DEseq (v1.28.1) [94] with a threshold of FDR < 0.05 and log2 (fold change) > 2. Co-expression gene networks were constructed by implementing organ DEGs using the R package WGCNA (v1.63) [95]. KEGG and GO enrichment analyses of each module in the networks were conducted using EnrichPipeline [91]. Cytoscape (v3.8.0) [96] was employed for the visualization of co-expression networks in the selected modules.

### Transcriptomic profiling under multiple environmental stresses

For aerial exposure, adult *M. mercenaria* were subjected to air in a thermostatic incubator at 15 °C and 50% humidity; aerial exposure lasting 16 days was found to be semi-lethal. We sampled the 3 replicates of clams (each replicate contains 3 individuals) on days 0, 8, and 16. For salinity challenge, adult *M. mercenaria* were subjected to different levels of salinity: 5, 15, 30, and 40 ppt for 10 days. Ten days were found to be semi-lethal for salinity at 5 ppt treatment. We sampled 3 replicates of live clams (each replicate contains 3 individuals) from each salinity treatment. For heat and hypoxia stress, adult *M. mercenaria* were subjected to heated seawater (35 °C) and normal seawater (20 °C) with DO (dissolved oxygen) at 0.2, 2, and 6 mg/L, respectively (2 × 3 treatment). We sampled 3 replicates of live clams (3 individuals in each replicate) on day 3 (semi-lethal at 35 °C and 0.2 mg/L DO) from each treatment. For all the above sampling, gill tissues were dissected with sterile scalpels for RNA extraction. Illumina sequencing, estimating of gene expression levels, and identification of DEGs were performed as described above. KEGG and GO enrichment analyses of DEGs were performed using the EnrichPipeline [91], and an R script was used to draw a volcano map of DEGs based on the enrichment results.

### Identification of the IAP gene family

Reference protein sequences of IAPs downloaded from NCBI and Uniprot databases were used for TBLASTN with e-value 1e-5 in the “-F F” option. High-quality BLAST hits that corresponded to reference proteins were concatenated via Solar software (v0.9.6) [68]. Sequence of each reference protein was extended upstream and downstream by 2000 bp to represent a protein-coding region. GeneWise software (v2.4.1) [69] was used to predict the exact gene structure of the corresponding genomic region of each BLAST hit. Using this process, candidate IAPs were identified; then, conserved domains and functional annotation of genes were identified via HMM search against the Pfam database and BLASTP against the non-redundant (nr) database. Finally, genes with BIR domains functionally annotated as IAPs in Nr-database were manually selected as the final identified products. Members of the IAP family were classified into different types based on the number and arrangement of conserved BIR and RING domains, which are the two core domains involved in mediating protein–protein interactions. Additionally, mafft software (v7.427) [97] was used to align protein sequences of IAPs from 19 species. The N-J method in TreeBest software (v1.9.2) [98] was used to construct the phylogenetic tree. Next, TBtools software (v0.665) was used to count and visualize the intron phase, distribution on chromosomes, character of domain conservation, and transcription direction of *M. mercenaria* IAPs based on gff3. Finally, the Ka and Ks of tandem IAPs from *M. mercenaria* were calculated using Calculator2.0 software [99]

To explore the impact of TEs in extensive expansion of IAP genes, we calculated TE density in the vicinity of genes in hard clam genome—10 kb upstream and downstream of each gene, separately for IAP genes and non-IAP genes. Statistical significance was assessed by *t* test. TE densities were analyzed separately for each TE types (DNA, LINE, LTR, SINE). To determine the evolutionary dynamics of the IAP family, we used the same method to identify the number of IAP family members in the 19 species subjected to phylogenetic analysis. Café software (v2.1) [90] was used to analyze the gain and loss of IAPs between these 19 species. Furthermore, IAPs from these 19 species were re-classified based on types.

### Phylostratigraphic analysis

We determined the time of origin of *M. mercenaria* IAPs and DEGs in selected organ modules. After these genes were obtained from WGCNA, they were first searched using BLASTP (*E*-value = 1e−10) against annotated proteins from the genomes of 21 species [100], with the first phylostratum (PS1) being the origin of cellular life (i.e., oldest genes), and the last phylostratum (PS13) being the lineage of the hard clam (newest genes). If one gene was identified in any of the 21 species, we assumed that the last common ancestor of that *M. mercenaria* gene, as well as respective species, already possessed a copy of this gene.

## Supplementary Information

**Additional file 1: Figure S1.** Ten IAPs in human genome.

**Additional file 2: Tables S1 to S9.** Background information for hard clam genome assembly and annotation. **Table S1**. Summary of sequencing data generated for the hard clam genome assembly. **Table S2.** Assembly results of the hard clam genome*.* **Table S3.** Hard clam genome characters estimated by *k*-mer analysis. **Table S4.** Hard clam genome assembly mapped to chromosomes. **Table S5.** Genomic read coverage statistics for the hard clam*.* **Table S6.** BUSCO assessment of the hard clam genome assembly. **Table S7.** Statistics of gene annotation and structural information. **Table S8.** Gene content and polymorphism in published bivalve and human genomes. **Table S9.** Classification of repetitive sequences and transposable elements in the hard clam genome.

**Additional file 3: Table S10.** List of gene families expanded in the hard clam genome.

**Additional file 4: Fig. S2.** HiC heatmap of 19 chromosomes of the hard clam genome.

**Additional file 5: Fig. S3.** KEGG enrichment of genes shown in brown, red, and turquoise modules.

**Additional file 6: Fig. S4.** Co-expression network analysis of genes enriched in top 20 KEGG pathways in the brown module.

**Additional file 7: Fig. S5.** Co-expression coefficient between IAPs clustered in the apoptosis pathway in **Fig. S4.** and other genes. A cut-off of > 0.35 (top 3%) was applied in WGCNA analysis to screen out strong correlation between IAPs and other genes. Line weight represents the correlation coefficient.

**Additional file 8: Table S11.** General properties of hard clam IAPs.

**Additional file 9: Fig. S6.** TE density in 10 kb windows around IAP and all genes in the hard clam genome.

**Additional file 10: Fig. S7.** Venn diagram of common and unique genes expressed in response to high temperature, hypoxia, low salinity and aerial exposure (left), and the Nr annotation of the 27 genes responded to all stressors.

**Additional file 11: Fig. S8.** Divergence in expression of IAPs from the brown module under multiple environmental stressors. (PreAE, pre-aerial exposure; PostAE8/16, 8/16 d post-aerial exposure; S_5/15/30/40, salinity 5/15/30/40 ppt, respectively; H_0.2/2/6, heated seawater 35 °C with DO at 0.2/2/6 mg/L, respectively; C_0.2/2/6, normal seawater 20 °C with DO at 0.2/2/6 mg/L, respectively).

**Additional file 12 Fig. S9.** Expression divergence of IAPs belonging to different clades in response to environmental stressors.

**Additional file 13 Fig. S10.** Expression divergence of IAPs belonging to different structural types in response to environmental stressors.

## Data Availability

All data generated or analyzed during this study are included in this published article and its supplementary information files. Reads produced in this study are available at the NCBI Short Read Archive (SRA) under accession PRJNA596049 [101] for reads used for the genome assembly and transcriptomic expression analysis. Other datasets (genome assembled sequence, official gene sets, gene models, transcriptome assembly) are available at the Figshare database [102].
