# Supplementary material for: The hard clam genome reveals massive expansion and diversification of inhibitors of apoptosis in Bivalvia
Source: BMC Biol. 2021 Jan 25;19:15. doi: 10.1186/s12915-020-00943-9 (PMC7831173; doi:10.1186/s12915-020-00943-9)

Accession number	Domain	Common name	Length	Structure types
NP_647478.1	PF00653.21:BIR PF13920.6:zf-C3HC4_3	ML-IAP	298aa	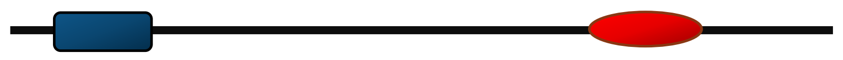
NP_203127.3	PF00653.21:BIR PF13920.6:zf-C3HC4_3	hILP-2	236aa	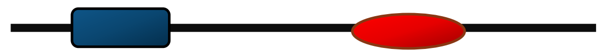
XP_005264506.2	PF00653.21:BIR PF12356.8:BIRC6 PF00179.26:UQ_con	Apollon	4884aa	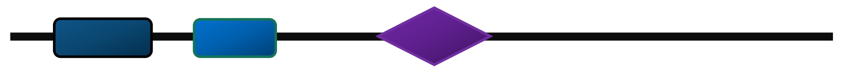
NP_001158.2	PF00653.21:BIR PF00653.21:BIR PF00653.21:BIR PF13920.6:zf-C3HC4_3	Xiap	497aa	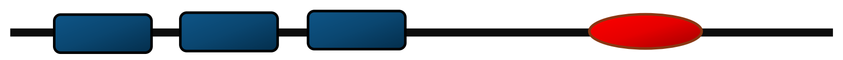
NP_001333799.1	PF00653.21:BIR PF00653.21:BIR PF00653.21:BIR PF05729.12:NACHT PF17779.1:NOD2_WH PF17889.1:NLRC4_HD	NAIP	1403aa	
NP_001333799.1_2	PF00653.21:BIR PF00653.21:BIR PF00653.21:BIR PF05729.12:NACHT PF17779.1:NOD2_WH PF17889.1:NLRC4_HD	NAIP	1403aa	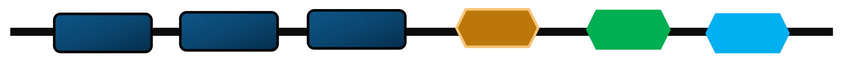
NP_001333799.1_3	PF00653.21:BIR PF00653.21:BIR PF00653.21:BIR PF05729.12:NACHT PF17779.1:NOD2_WH PF17889.1:NLRC4_HD	NAIP	1403aa	
NP_001156.1	PF00653.21:BIR PF00653.21:BIR PF00653.21:BIR PF00619.21:CARD PF13920.6:zf-C3HC4_3	c-IAP2	604aa	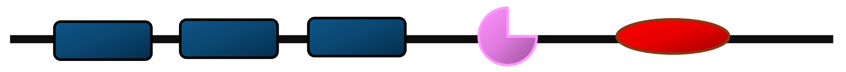
NP_001157.1	PF00653.21:BIR PF00653.21:BIR PF00653.21:BIR PF00619.21:CARD PF13920.6:zf-C3HC4_3	c-IAP1	618aa	
NP_001012271.1	PF00653.21:BIR	survivin	165aa	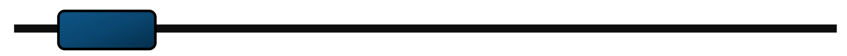

Key to domains

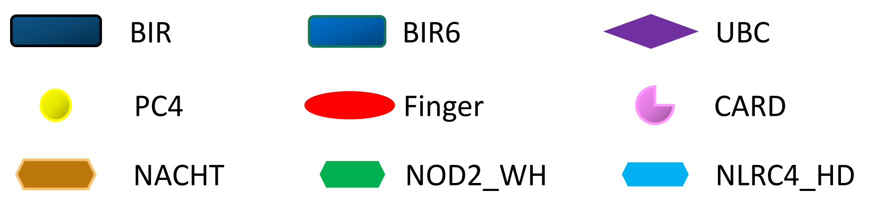

Supplement: Supplementary file 1 — Additional file 1: Figure S1. Ten IAPs in human genome. [file 12915_2020_943_MOESM1_ESM.pdf]
