# Supplementary material for: The hard clam genome reveals massive expansion and diversification of inhibitors of apoptosis in Bivalvia
Source: BMC Biol. 2021 Jan 25;19:15. doi: 10.1186/s12915-020-00943-9 (PMC7831173; doi:10.1186/s12915-020-00943-9)
Supplement: Supplementary file 2 — Additional file 2: Tables S1 to S9. Background information for hard clam genome assembly and annotation. Table S1. Summary of sequencing data generated for the hard clam genome assembly. Table S2. Assembly results of the hard clam genome. Table S3. Hard clam genome characters estimated by k-mer analysis. Table S4. Hard clam genome assembly mapped to chromosomes. Table S5. Genomic read coverage statistics for the hard clam. Table S6. BUSCO assessment of the hard clam genome assembly. Table S7. Statistics of gene annotation and structural information. Table S8. Gene content and polymorphism in published bivalve and human genomes. Table S9. Classification of repetitive sequences and transposable elements in the hard clam genome. [file 12915_2020_943_MOESM2_ESM.docx]

Table S1. Summary of sequencing data generated for the hard clam genome assembly.

| **Pair-end libraries** | **Insert size** | **Total data (G)** | **Read length (bp)** | **Sequence coverage (X)** |
| --- | --- | --- | --- | --- |
| **Illumina reads** | 350bp | 422.93 | 150 | 238.07 |
| **PacBio reads** | - | 179.74 | - | 101.18 |
| **10X Genomics** | - | 190.14 | - | 107.03 |
| **Hi-C** | - | 193.74 | 150 | 108.84 |
| **Total** | - | 986.55 | - | 555.12 |

Table S2. Assembly results of the hard clam genome.

| Sample ID | Length | | Number | |
| --- | --- | --- | --- | --- |
|  | Contig**(bp) | Scaffold(bp) | Contig** | Scaffold |
| Total | 1,778,824,901 | 1,788,414,051 | 4,223 | 1,541 |
| Max | 14,244,866 | 126,482,403 | - | - |
| Number≥2000 | - | - | 4,222 | 1,541 |
| N50 | 1,768,861 | 91379,220 | 262 | 9 |
| N60 | 1,327,074 | 90,138,423 | 377 | 11 |
| N70 | 918,953 | 86,026,883 | 539 | 13 |
| N80 | 548,380 | 82,308,154 | 788 | 15 |
| N90 | 241,845 | 75,297,454 | 1,271 | 17 |

Table S3. Hard clam genome characters estimated by *k*-mer analysis.

| *k* -mer | *k*-mer number | *k* -mer Depth | Genome  Size (Mbp) | Revised Genome Size (Mbp) | Heterozygous Ratio (%) | Repeat (%) |
| --- | --- | --- | --- | --- | --- | --- |
| 17 | 95,219,358,284 | 53 | 1,796.59 | 1,776.51 | 1.34 | 63.31 |

Table S4. Hard clam genome assembly mapped to chromosomes.

| Chromosome | Mapped length |
| --- | --- |
| Chr_1 | 86026883 |
| Chr_2 | 125760036 |
| Chr_3 | 92285503 |
| Chr_4 | 91379220 |
| Chr_5 | 108648503 |
| Chr_6 | 87328355 |
| Chr_7 | 110078379 |
| Chr_8 | 96626343 |
| Chr_9 | 126482403 |
| Chr_10 | 93586546 |
| Chr_11 | 98843772 |
| Chr_12 | 90138423 |
| Chr_13 | 82308154 |
| Chr_14 | 90607396 |
| Chr_15 | 85354191 |
| Chr_16 | 76630363 |
| Chr_17 | 70645253 |
| Chr_18 | 75297454 |
| Chr_19 | 49502607 |
| Chr_total | 1737529784 |
| **Chromosome mapping ratio** | **0.971547826** |

Fig S5. Genomic reads coverage statistics for the hard clam*.*

|  |  | Percentage |
| --- | --- | --- |
| Reads | Mapping rate (%) | 95.28 |
|  | Average sequencing depth | 64.20 |
| Genome | Coverage (%) | 98.00 |
|  | Coverage at least 4X (%) | 97.69 |
|  | Coverage at least 10X (%) | 97.39 |
|  | Coverage at least 20X (%) | 96.62 |

Mapping rate: Rate of reads mapping to genomes.

Average sequence depth：The average depth of each base on the genome covered by reads.

Coverage：Proportion of genome covered by reads.

Coverage at least NX(%)：Proportion of genome covered by NX reads.

Fig S6. BUSCO assessment of the hard clam genome assembly.

| BUSCO |
| --- |
| 90.5% [S:88.4%, D:2.1%], F:0.9%, M:8.6%, n:954 |

Table S7. Statistics of gene annotation and structure information.

|  | Gene set | Number | Average transcript length(bp) | Average CDS length(bp) | Average exons per gene | Average exon length(bp) | Average intron length(bp) |
| --- | --- | --- | --- | --- | --- | --- | --- |
| De novo | Augustus | 57,124 | 10,539.94 | 1,159.37 | 4.44 | 261.37 | 2,730.32 |
|  | GlimmerHMM | 313,215 | 4,898.32 | 418.09 | 2.51 | 166.25 | 2,957.50 |
|  | SNAP | 61,593 | 5,971.75 | 479.28 | 2.96 | 161.69 | 2,796.19 |
|  | Geneid | 32,339 | 39,773.72 | 573.28 | 5.77 | 99.32 | 8,214.40 |
|  | Genscan | 49,244 | 22,751.06 | 1,379.17 | 5.21 | 264.96 | 5,082.35 |
| Homolog | Bfl | 34,322 | 3,603.85 | 637.63 | 2.25 | 283.9 | 2,380.66 |
|  | Bpl | 66,583 | 3,069.63 | 815.42 | 2.2 | 370.36 | 1,875.85 |
|  | Cfa | 33,813 | 5,940.80 | 853.99 | 3.09 | 276.54 | 2,436.06 |
|  | Cvi | 45,526 | 4,986.11 | 1,167.68 | 2.9 | 402.56 | 2,009.01 |
|  | Lgi | 71,264 | 2,721.23 | 623.69 | 2.03 | 307.14 | 2,035.17 |
|  | Mph | 62,335 | 2,935.49 | 825.19 | 2.2 | 374.86 | 1,756.60 |
|  | Obi | 32,366 | 4,397.53 | 792.64 | 2.56 | 309.38 | 2,307.86 |
|  | Pca | 31,003 | 5,783.23 | 991.84 | 3.05 | 325.2 | 2,337.27 |
|  | Pfu | 42,887 | 4,475.31 | 1,042.38 | 2.67 | 390.99 | 2,060.57 |
|  | Pye | 53,191 | 4,370.70 | 1,028.88 | 2.7 | 381.24 | 1,967.23 |
| RNAseq | PASA | 46,806 | 15,437.14 | 1,050.23 | 4.94 | 212.49 | 3,649.20 |
|  | Cufflinks | 88,239 | 24,562.72 | 2,902.93 | 6.6 | 439.97 | 3,869.18 |
| EVM |  | 47,234 | 11,326.32 | 1,085.47 | 4.71 | 230.6 | 2,762.46 |
| Pasa-update* | | 46,422 | 12,137.48 | 1,120.21 | 4.86 | 230.45 | 2,853.44 |
| Final set** |  | 34,283 | 15,547.97 | 1,219.96 | 6.04 | 202.07 | 2,844.40 |

Table S8. Gene content and polymorphism of published bivalve and human genomes.

| **Species** | **Gene No.** | **Polymorphism** | **Reference** |
| --- | --- | --- | --- |
| *Mercenaria mercenaria* | 34283 | 0.0134 | this study |
| *Crassostrea gigas* | 28398 | 0.0130 | Zhang et al. 2012 |
| *Crassostrea virginica* | 34596 | 0.0181 | Warren et al. 2018 |
| *Saccosstrea glomerata* | 29738 | 0.0051 | Powell et al. 2018 |
| *Pinctada martensii* | 32937 | 0.0140 | Du et al. 2017 |
| *Modiolus philippinarum* | 36549 | 0.0202 | Sun et al. 2017 |
| *Bathymodiolus platifrons* | 33584 | 0.0124 | Sun et al. 2017 |
| *Mizuhopecten yessoensis* | 26415 | 0.0104 | Wang et al. 2017 |
| *Azumapecten farreri* | 28602 | 0.0177 | Li et al. 2017 |
| *Ruditapes philippinarum* | 27652 | 0.0169 | Yan et al. 2019 |
| *Sinonovacula constricta* | 28594 | 0.0155 | Dong et al. 2019 |
| *Homo sapiens* | 20433 | 0.0013 | 100 GPC 2010 |

Table S9. Classification of repetitive sequence and transposable elements in hard clam genome.

| **Repetitive sequence** | | | **Transposable elements** | | |
| --- | --- | --- | --- | --- | --- |
| **Type** | Length (Bp) | % in genome | **Type** | Length (Bp) | % in genome |
| **DNA** | 612265540 | 34.235111 | **DNA** | 612265540 | 34.23511 |
| **LINE** | 128169576 | 7.166661 | **LINE** | 128169576 | 7.166661 |
| **SINE** | 7265695 | 0.406265 | **SINE** | 7265695 | 0.406265 |
| **LTR** | 179519065 | 10.037892 | **LTR** | 179519065 | 10.03789 |
| **Satellite** | 2116990 | 0.118372 | **Total** | 878334151 | 49.11246 |
| **Simple_repeat** | 10976509 | 0.613757 |  |  |  |
| **Unknown** | 50054259 | 2.798807 |  |  |  |
| **Total** | 930397817 | 52.023625 |  |  |  |

1. Dong, Y., Zeng, Q., Ren, J., Yao, H., Ruan, W., Lv, L., . . . Lin, Z. (2019). doi:10.1101/735142
2. Du, X., Fan, G., Jiao, Y., Zhang, H., Guo, X., Huang, R., . . . Liu, X. (2017). The pearl oyster Pinctada fucata martensii genome and multi-omic analyses provide insights into biomineralization. *Gigascience, 6*(8), 1-12. doi:10.1093/gigascience/gix059
3. Li, Y., Sun, X., Hu, X., Xun, X., Zhang, J., Guo, X., . . . Bao, Z. (2017). Scallop genome reveals molecular adaptations to semi-sessile life and neurotoxins. *Nat Commun, 8*(1), 1721. doi:10.1038/s41467-017-01927-0
4. Powell, D., Subramanian, S., Suwansa-Ard, S., Zhao, M., O'Connor, W., Raftos, D., & Elizur, A. (2018). The genome of the oyster Saccostrea offers insight into the environmental resilience of bivalves. *DNA Res, 25*(6), 655-665. doi:10.1093/dnares/dsy032
5. Sun, J., Zhang, Y., Xu, T., Zhang, Y., Mu, H., Zhang, Y., . . . Qian, P. Y. (2017). Adaptation to deep-sea chemosynthetic environments as revealed by mussel genomes. *Nat Ecol Evol, 1*(5), 121. doi:10.1038/s41559-017-0121
6. Wang, S., Zhang, J., Jiao, W., Li, J., Xun, X., Sun, Y., . . . Bao, Z. (2017). Scallop genome provides insights into evolution of bilaterian karyotype and development. *Nat Ecol Evol, 1*(5), 120. doi:10.1038/s41559-017-0120
7. Yan, X., Nie, H., Huo, Z., Ding, J., Li, Z., Yan, L., . . . Li, D. (2019). Clam Genome Sequence Clarifies the Molecular Basis of Its Benthic Adaptation and Extraordinary Shell Color Diversity. *iScience, 19*, 1225-1237. doi:10.1016/j.isci.2019.08.049
8. Zhang, G., Fang, X., Guo, X., Li, L., Luo, R., Xu, F., . . . Wang, J. (2012). The oyster genome reveals stress adaptation and complexity of shell formation. *Nature, 490*(7418), 49-54. doi:10.1038/nature11413
9. Warren, W., Guo, X., Proestou, D.A., Minx, P., Tomlinson, C. and Gomez-Chiarri, M., The Eastern Oyster Genome: A Resource for Comparative Genomics in Shellfish Aquaculture Species. In *Plant and Animal Genome XXVI Conference (January 13-17, 2018)*, p0280. https://pag.confex.com/pag/xxvi/meetingapp.cgi/Paper/29827
