# Supplementary figures and images for: The hard clam genome reveals massive expansion and diversification of inhibitors of apoptosis in Bivalvia
Source: BMC Biol. 2021 Jan 25;19:15. doi: 10.1186/s12915-020-00943-9 (PMC7831173; doi:10.1186/s12915-020-00943-9)

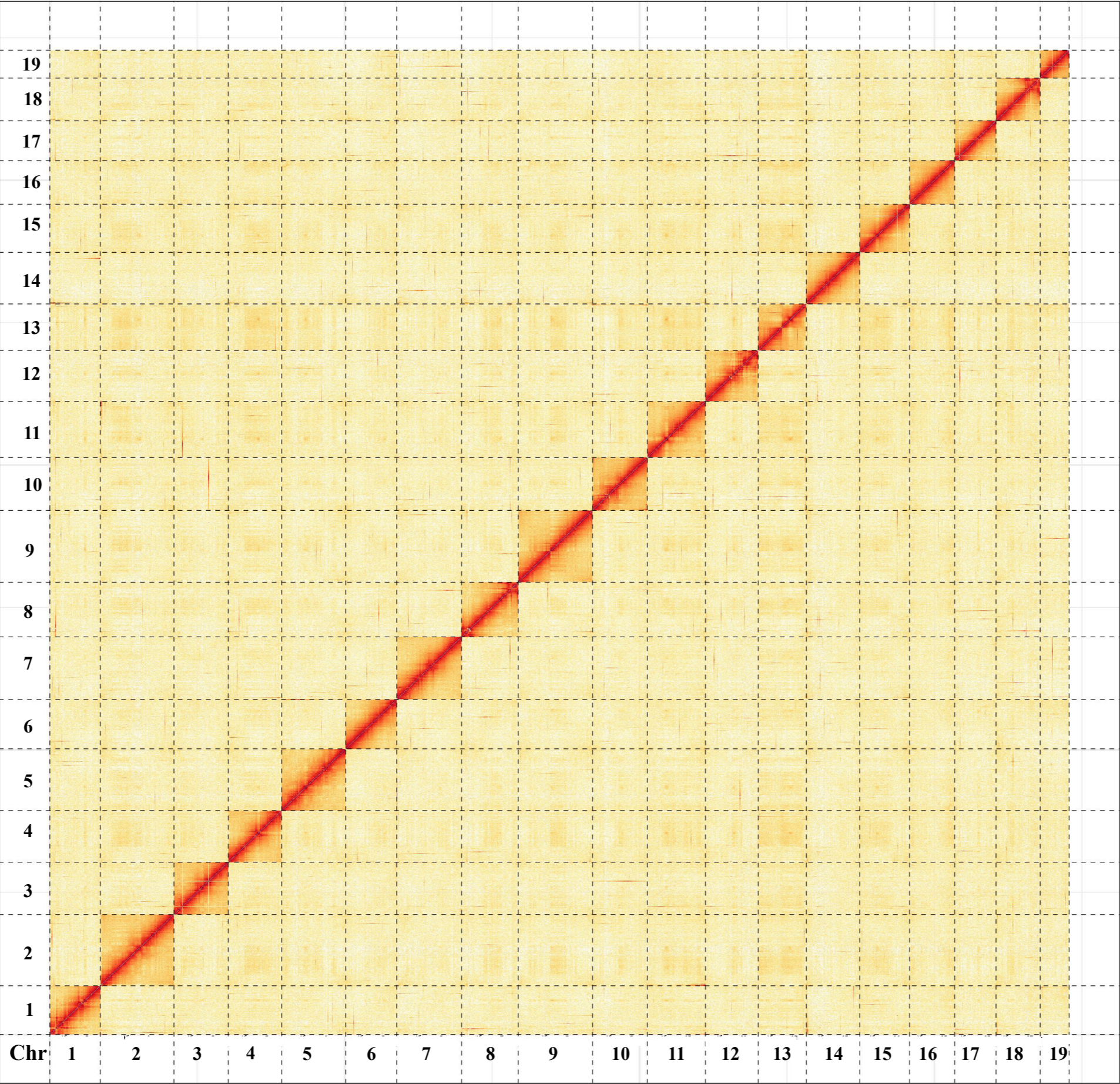

Supplement: Supplementary file 4 — Additional file 4: Fig. S2. HiC heatmap of 19 chromosomes of the hard clam genome. [file 12915_2020_943_MOESM4_ESM.pdf]

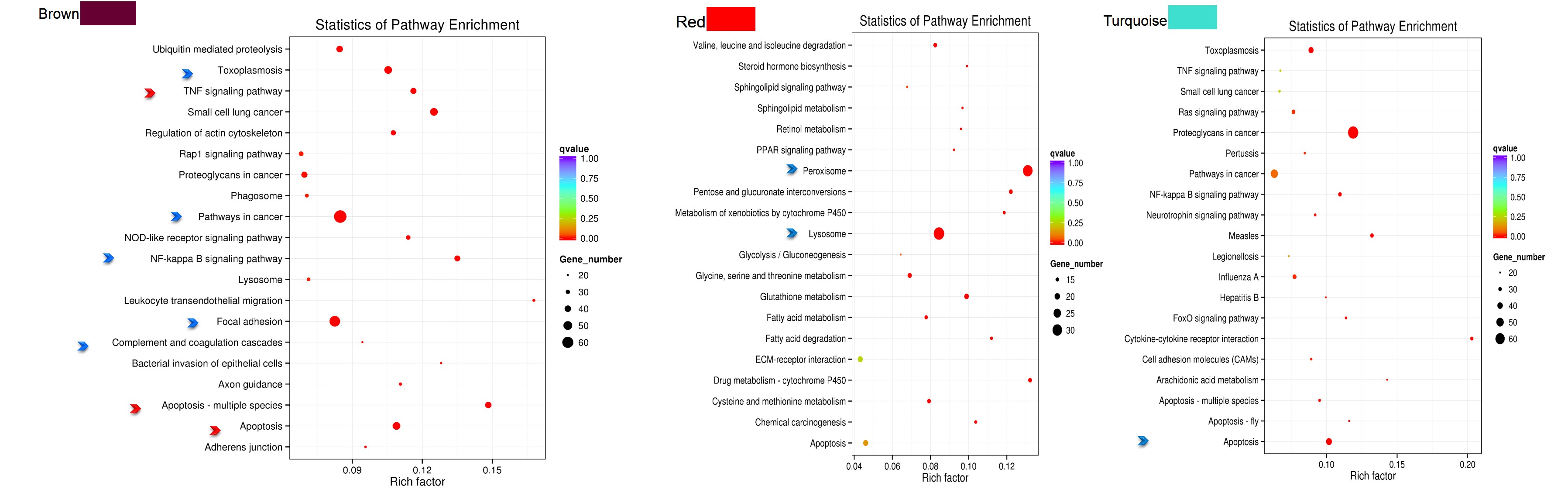

Supplement: Supplementary file 5 — Additional file 5: Fig. S3. KEGG enrichment of genes shown in brown, red, and turquoise modules. [file 12915_2020_943_MOESM5_ESM.jpg]

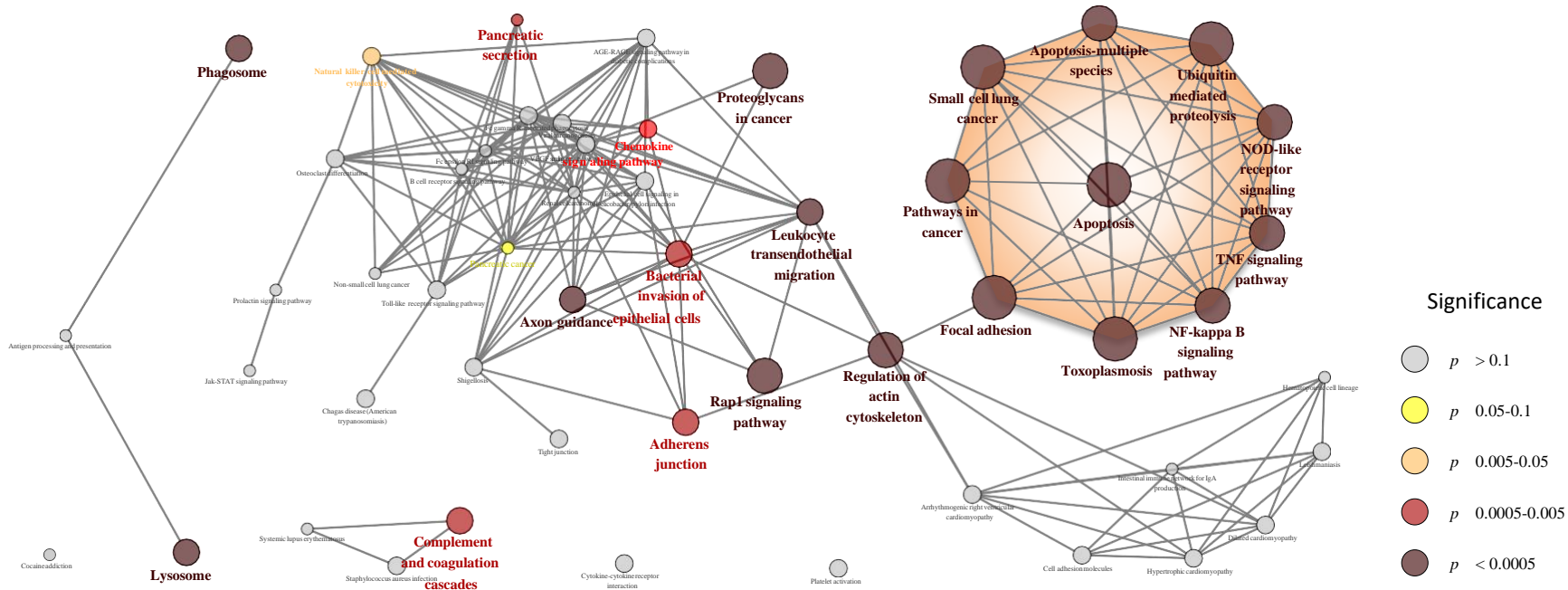

Supplement: Supplementary file 6 — Additional file 6: Fig. S4. Co-expression network analysis of genes enriched in top 20 KEGG pathways in the brown module. [file 12915_2020_943_MOESM6_ESM.pdf]

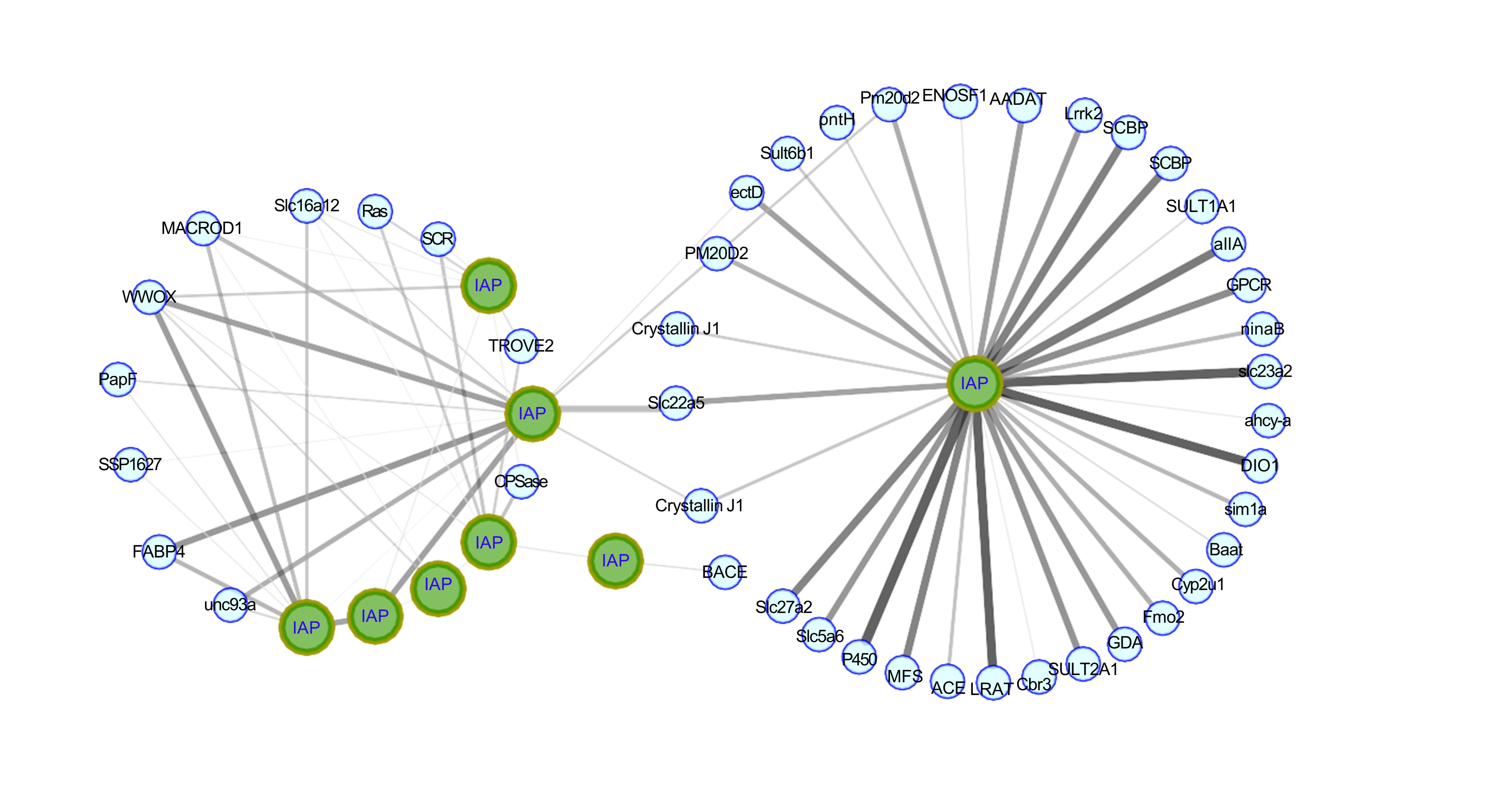

Supplement: Supplementary file 7 — Additional file 7: Fig. S5. Co-expression coefficient between IAPs clustered in the apoptosis pathway in Fig. S4. and other genes. A cut-off of > 0.35 (top 3%) was applied in WGCNA analysis to screen out strong correlation between IAPs and other genes. Line weight represents the correlation coefficient. [file 12915_2020_943_MOESM7_ESM.jpg]

# TE density

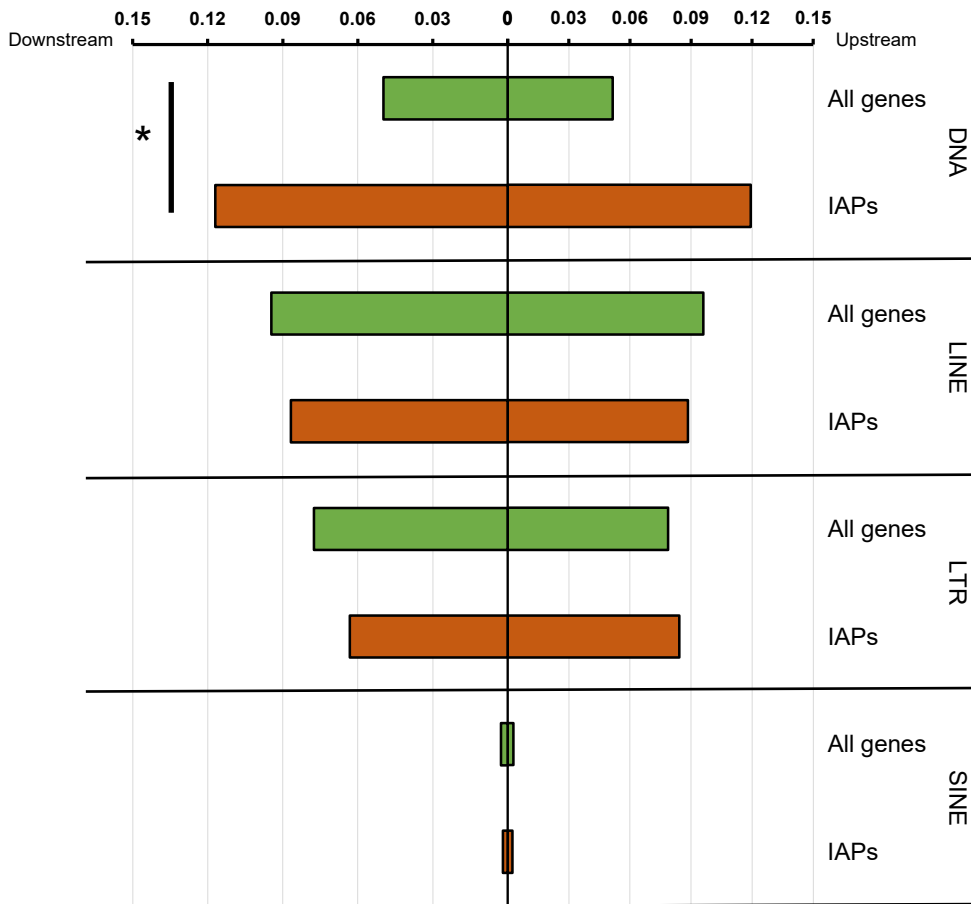

Supplement: Supplementary file 9 — Additional file 9: Fig. S6. TE density in 10 kb windows around IAP and all genes in the hard clam genome. [file 12915_2020_943_MOESM9_ESM.pdf]

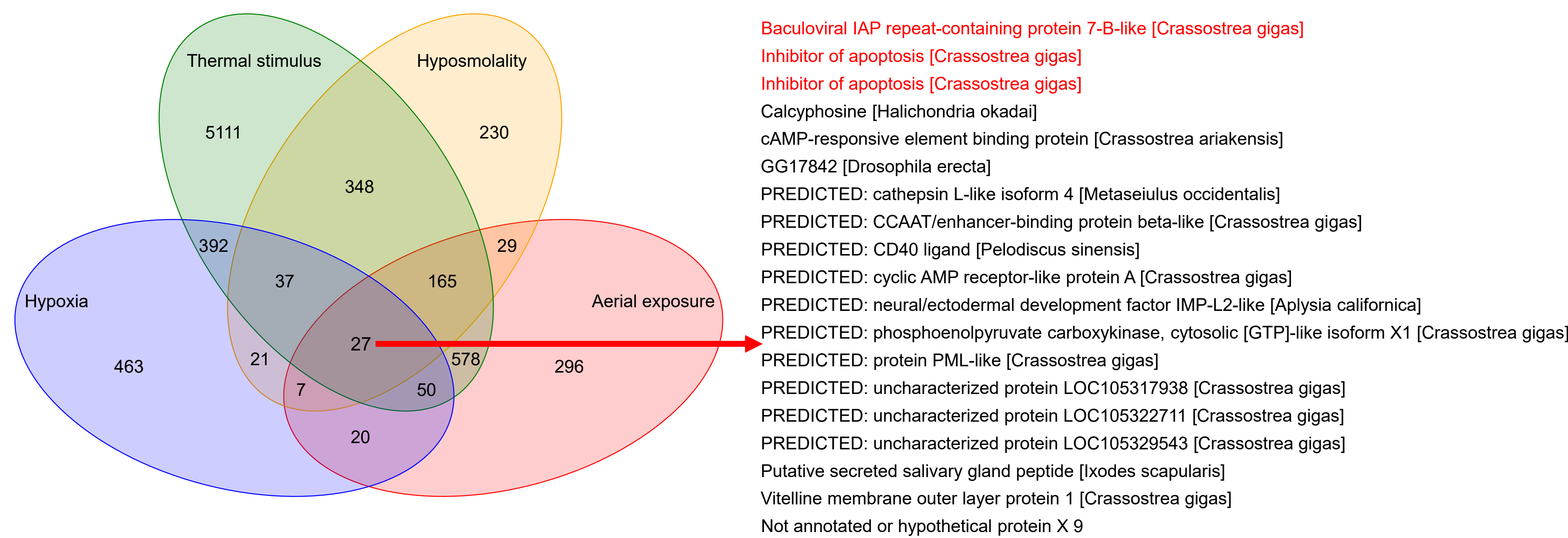

Supplement: Supplementary file 10 — Additional file 10: Fig. S7. Venn diagram of common and unique genes expressed in response to high temperature, hypoxia, low salinity and aerial exposure (left), and the Nr annotation of the 27 genes responded to all stressors. [file 12915_2020_943_MOESM10_ESM.pdf]

Gene expression divergence of IAPs in brown module

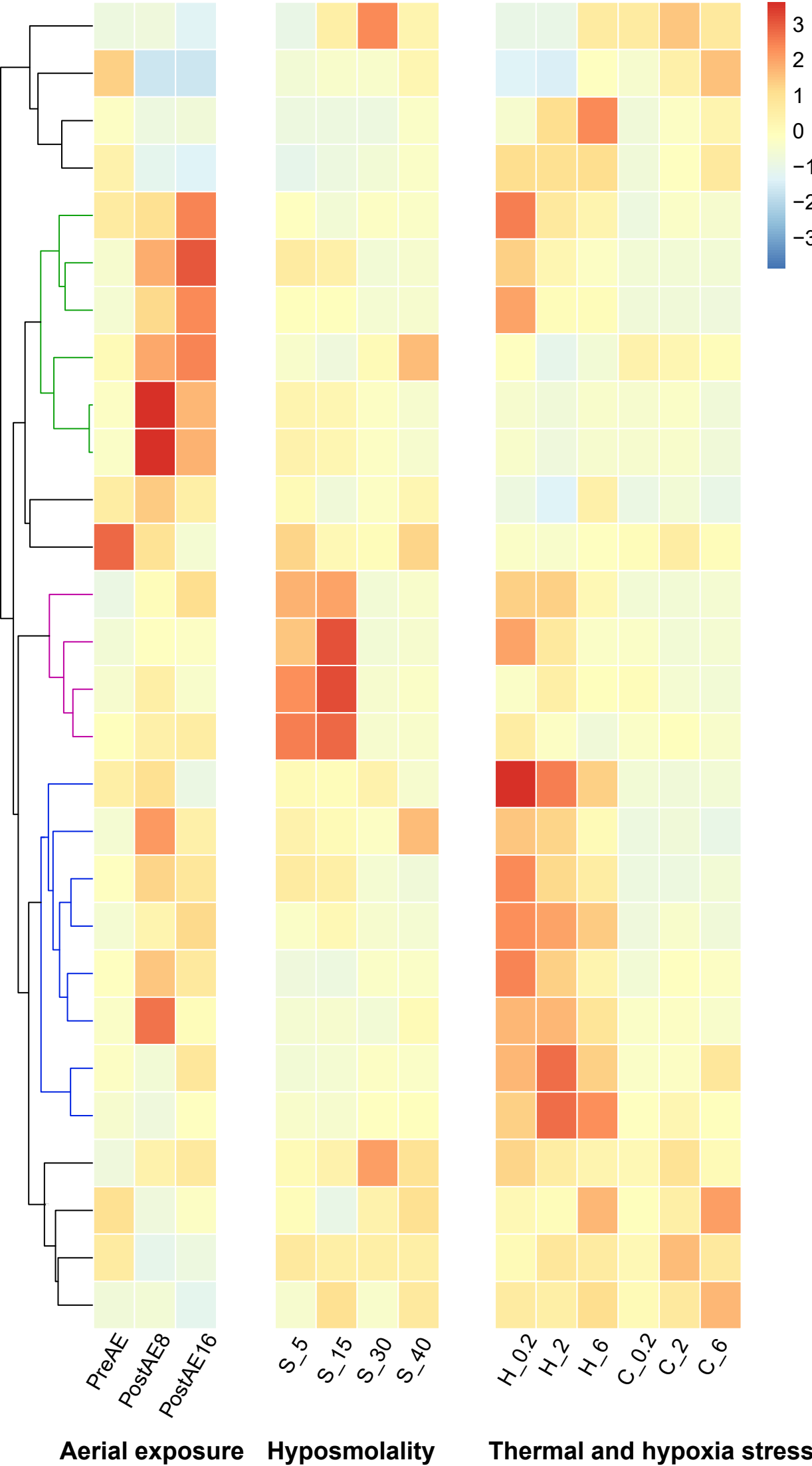

Supplement: Supplementary file 11 — Additional file 11: Fig. S8. Divergence in expression of IAPs from the brown module under multiple environmental stressors. (PreAE, pre-aerial exposure; PostAE8/16, 8/16 d post-aerial exposure; S_5/15/30/40, salinity 5/15/30/40 ppt, respectively; H_0.2/2/6, heated seawater 35 °C with DO at 0.2/2/6 mg/L, respectively; C_0.2/2/6, normal seawater 20 °C with DO at 0.2/2/6 mg/L, respectively). [file 12915_2020_943_MOESM11_ESM.pdf]

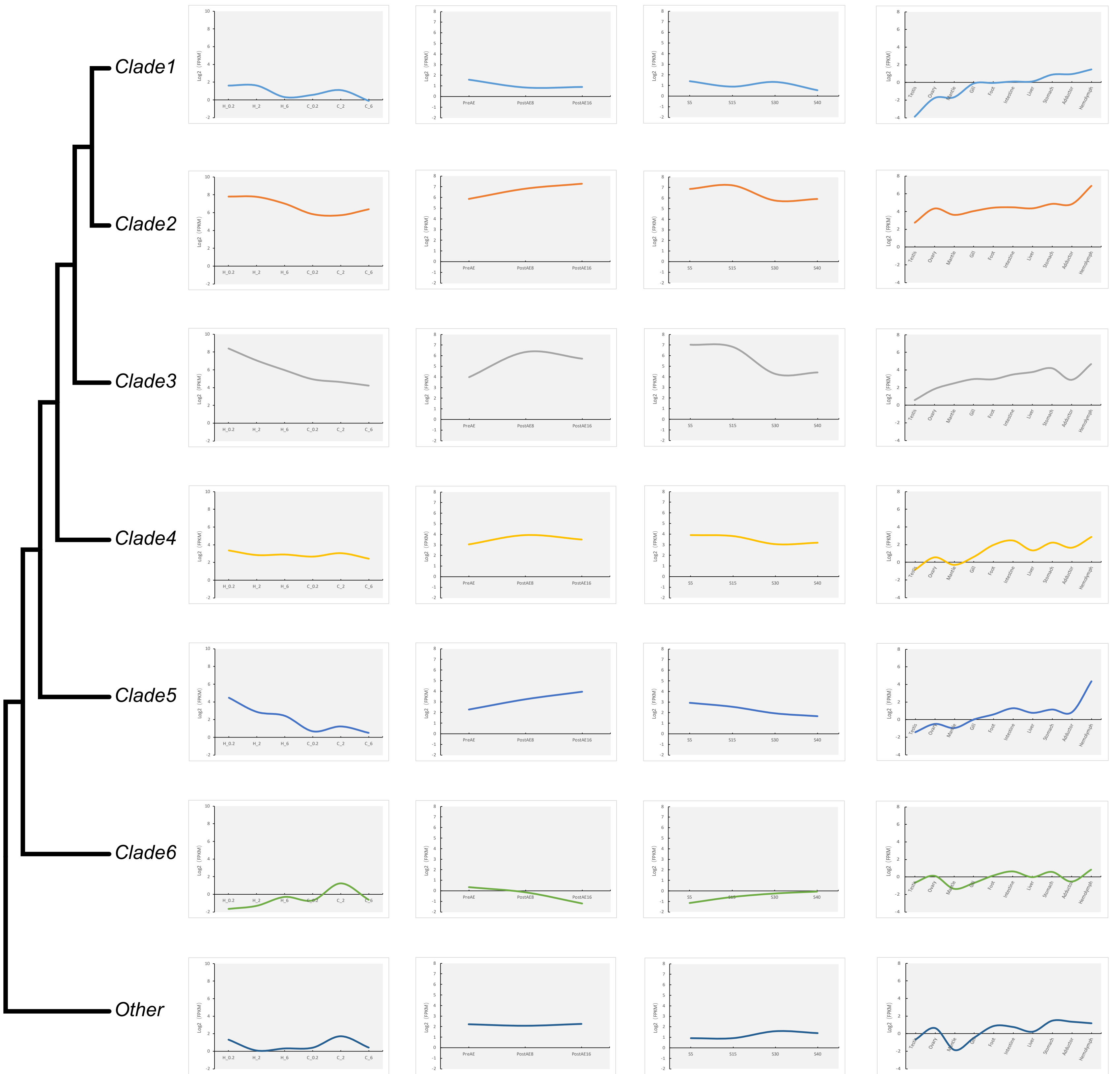

Supplement: Supplementary file 12 — Additional file 12 Fig. S9. Expression divergence of IAPs belonging to different clades in response to environmental stressors. [file 12915_2020_943_MOESM12_ESM.pdf]

**A**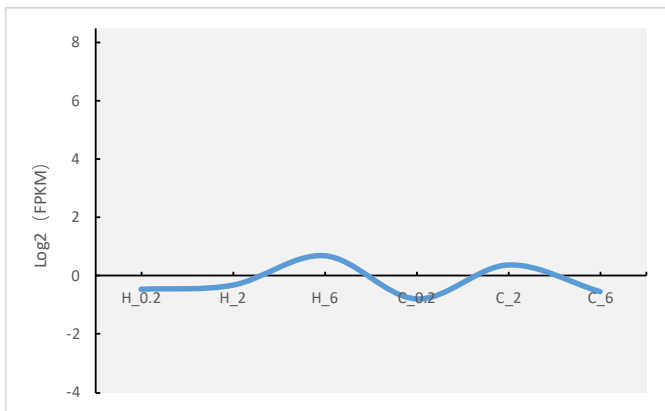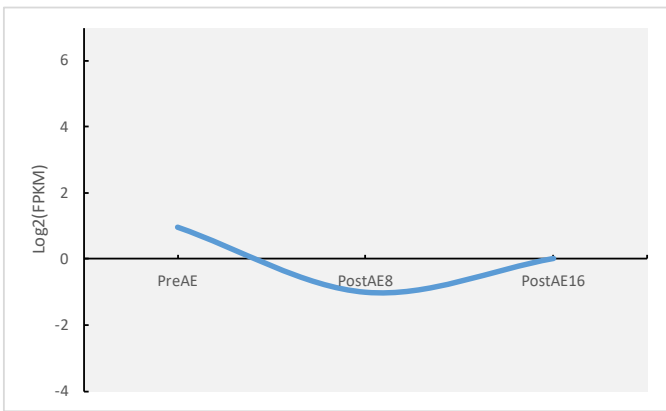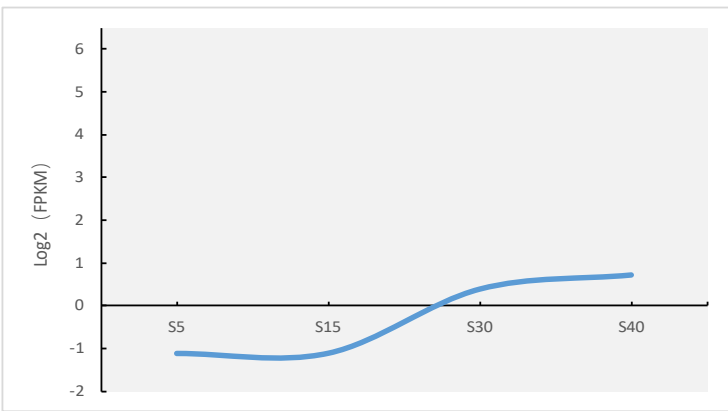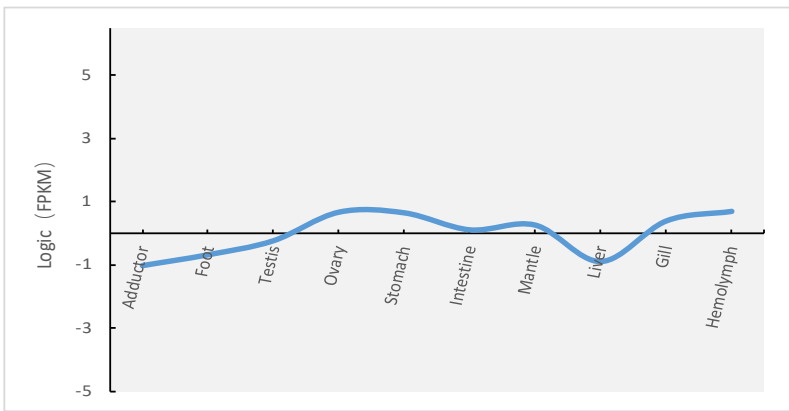**B**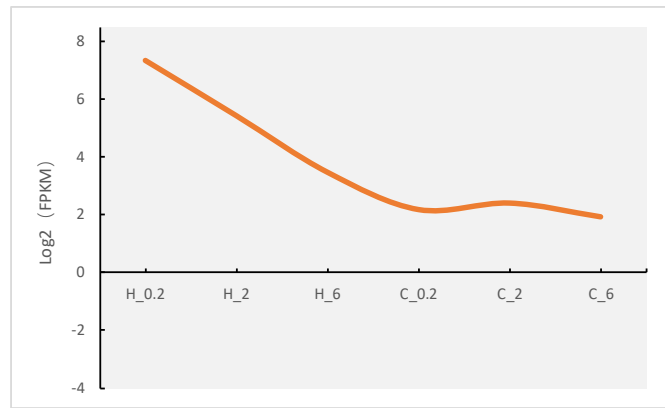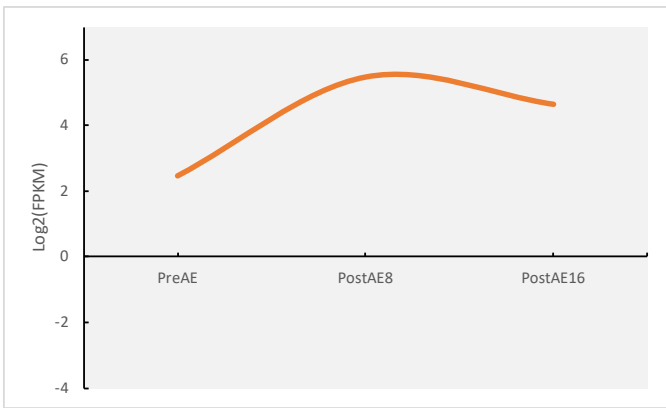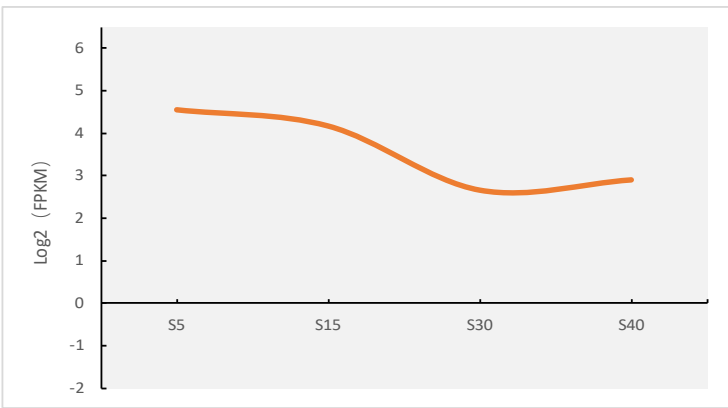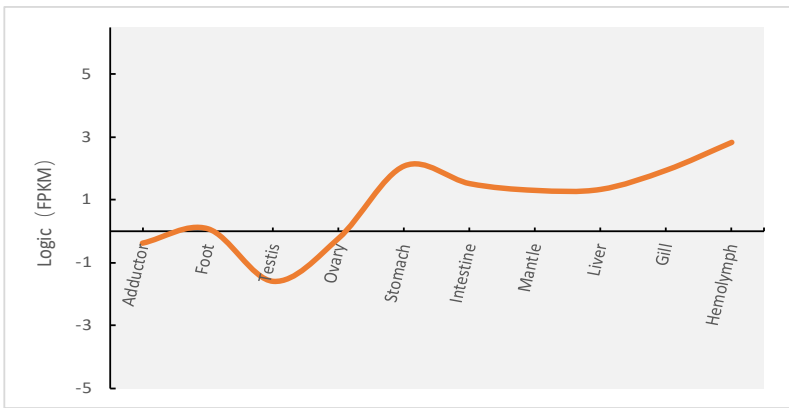**C**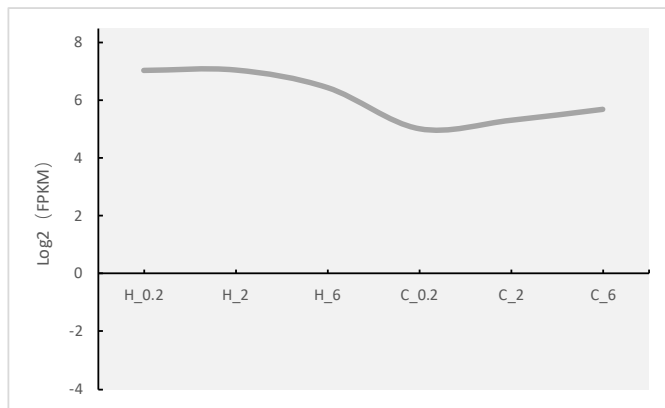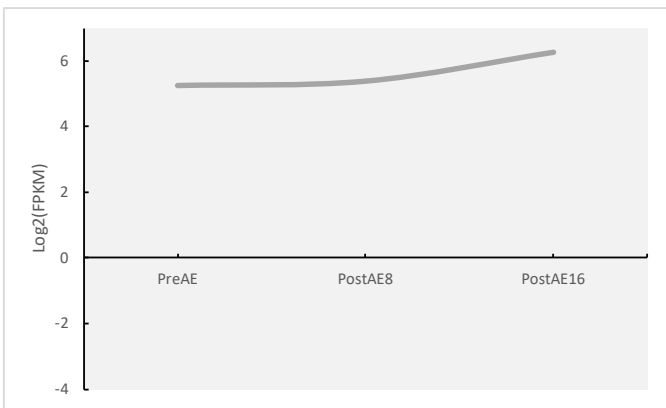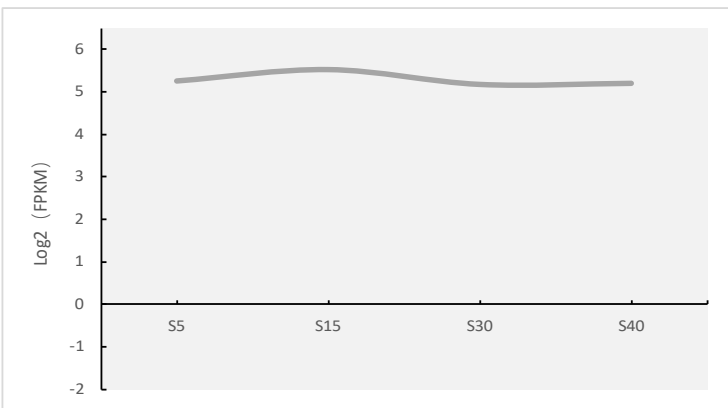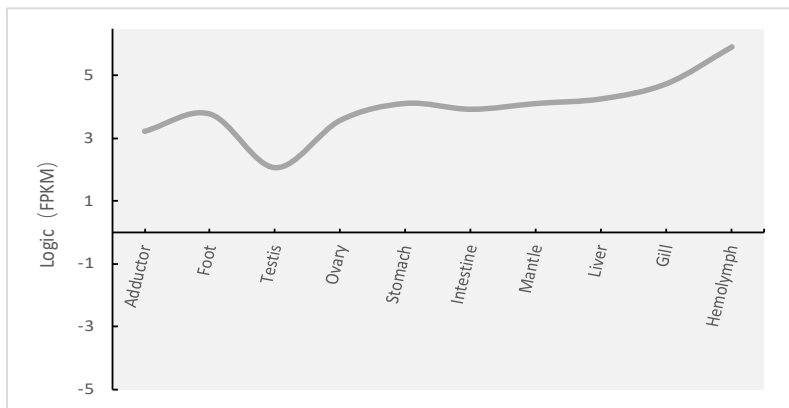**D**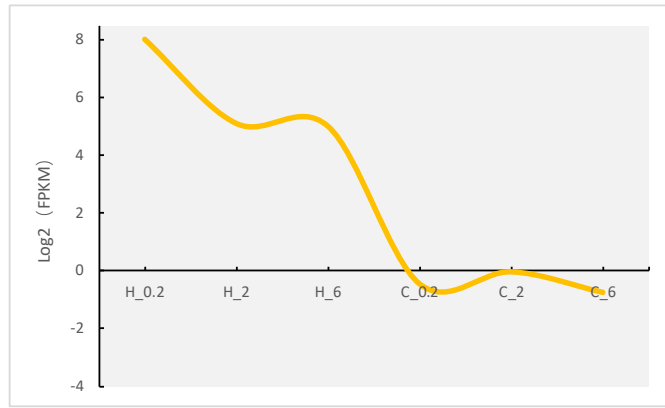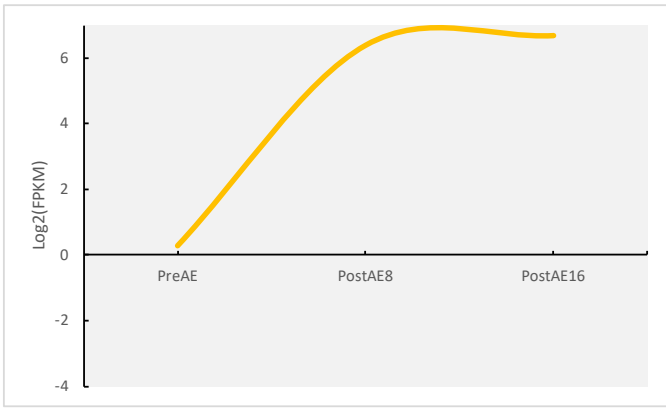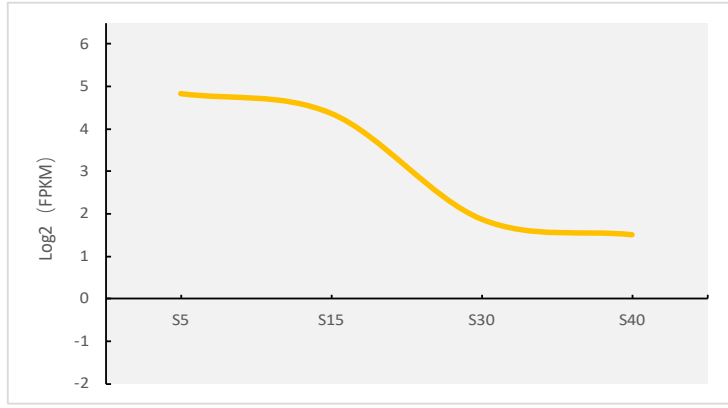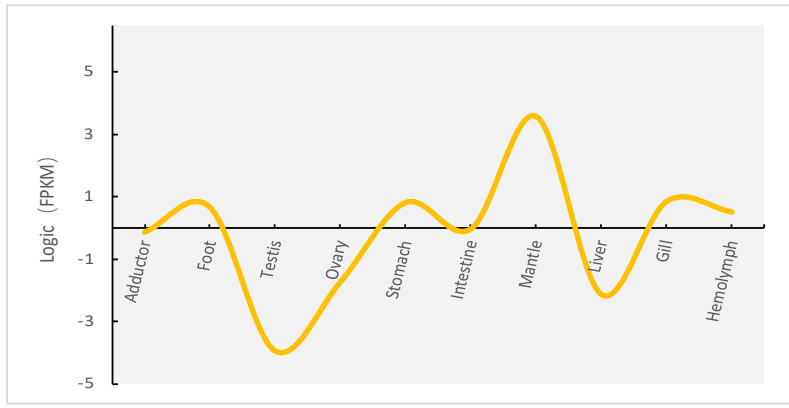**E**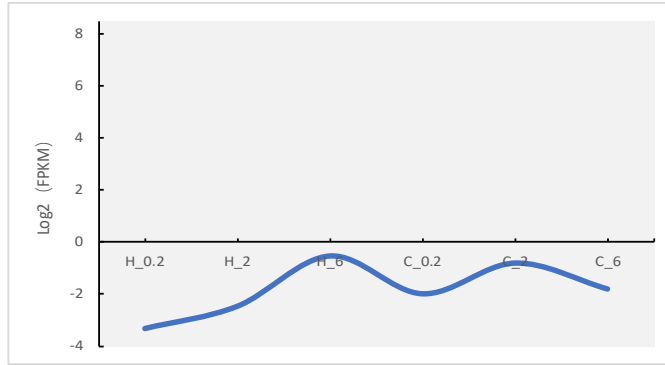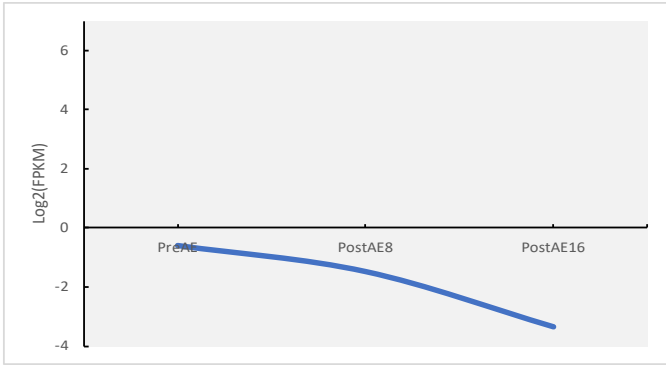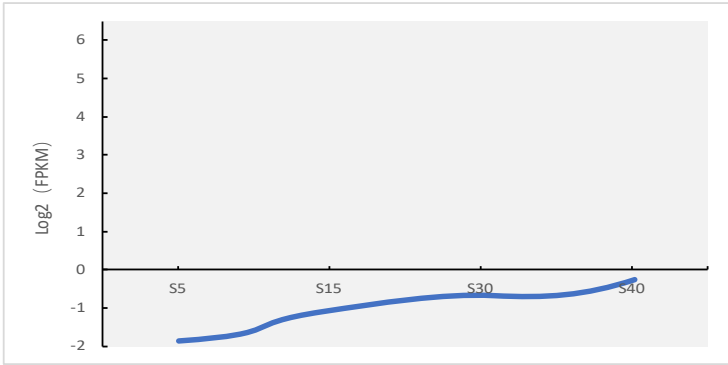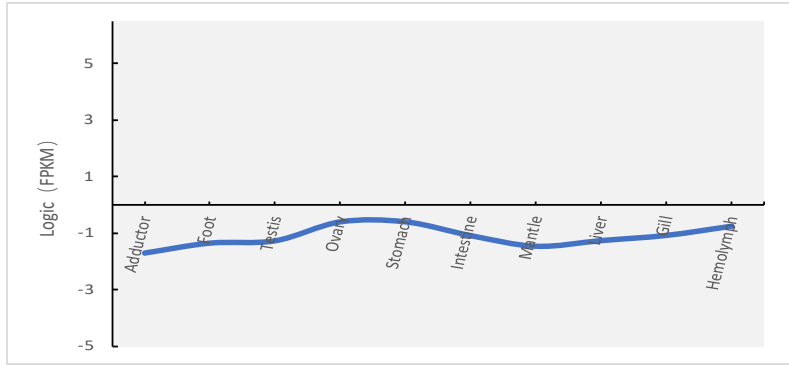**F**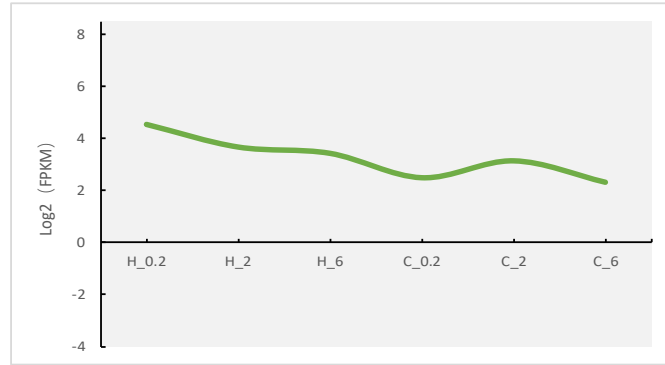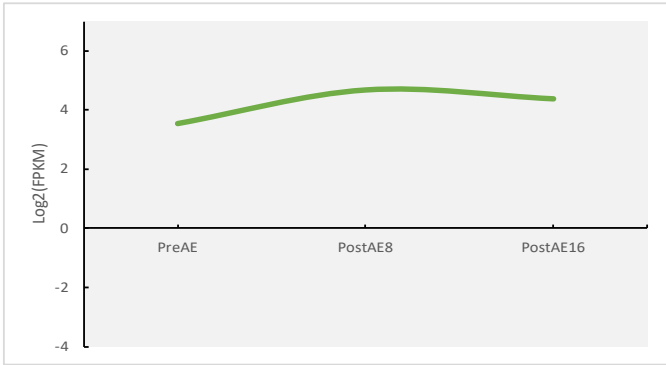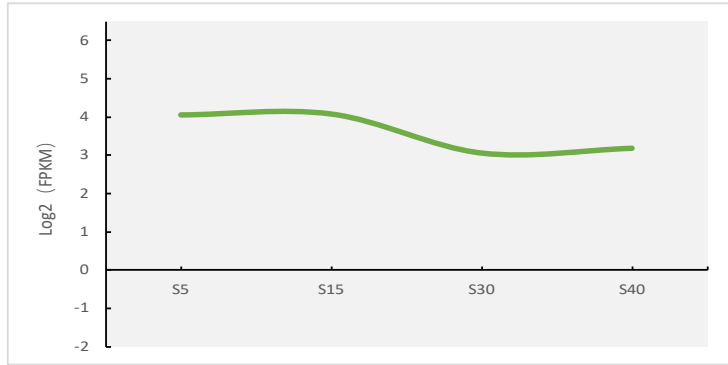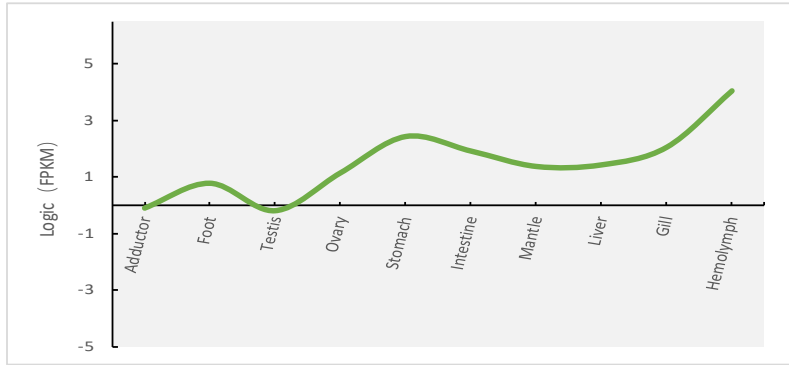**G**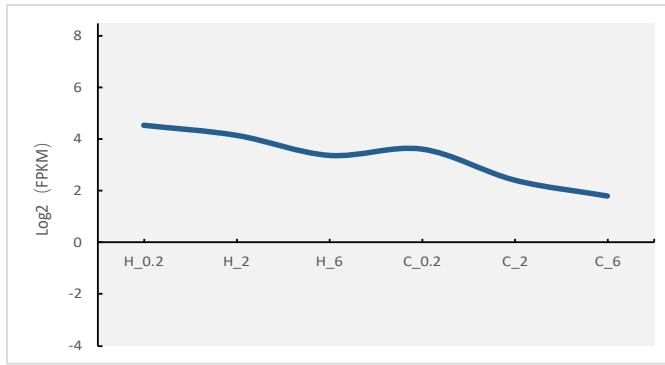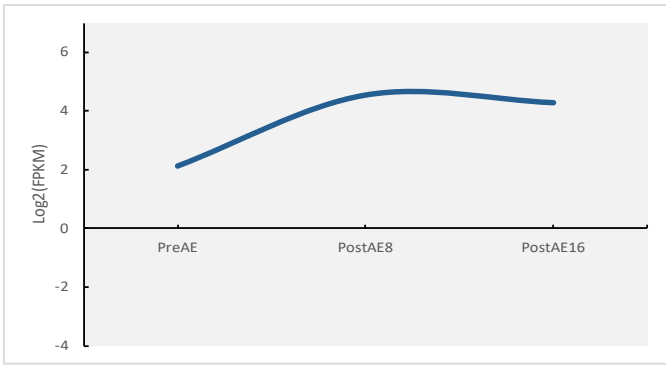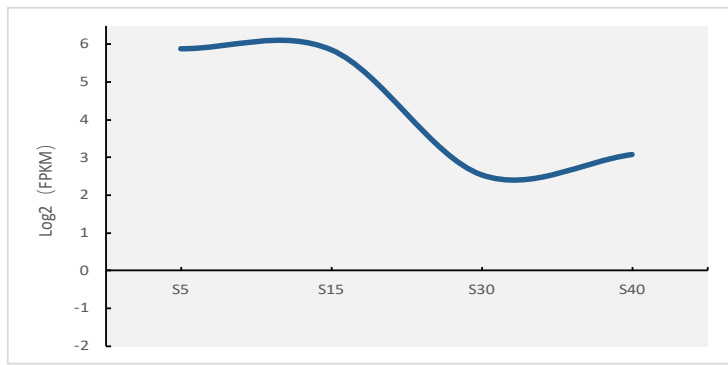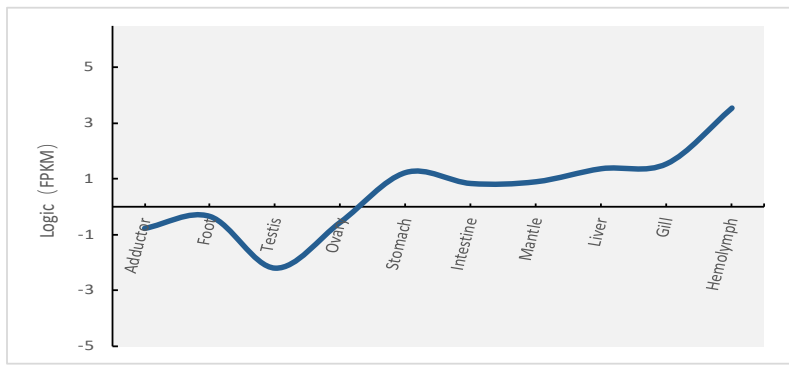

Supplement: Supplementary file 13 — Additional file 13 Fig. S10. Expression divergence of IAPs belonging to different structural types in response to environmental stressors. [file 12915_2020_943_MOESM13_ESM.pdf]
